# Characterisation of the Function of a SINE-VNTR-*Alu* Retrotransposon to Modulate Isoform Expression at the *MAPT* Locus

**DOI:** 10.3389/fnmol.2022.815695

**Published:** 2022-03-09

**Authors:** Alexander Fröhlich, Abigail L. Pfaff, Vivien J. Bubb, Sulev Koks, John P. Quinn

**Affiliations:** ^1^Department of Pharmacology and Therapeutics, Institute of Systems, Molecular and Integrative Biology, University of Liverpool, Liverpool, United Kingdom; ^2^Centre for Molecular Medicine and Innovative Therapeutics, Murdoch University, Perth, WA, Australia; ^3^Perron Institute for Neurological and Translational Science, Perth, WA, Australia

**Keywords:** transposable elements, MAPT, tau, Parkinson’s disease, ALS, FTD, motor neuron disease, SVA

## Abstract

SINE-VNTR-*Alu* retrotransposons represent one class of transposable elements which contribute to the regulation and evolution of the primate genome and have the potential to be involved in genetic instability and disease progression. However, these polymorphic elements have not been extensively analysed when addressing the missing heritability of neurodegenerative diseases, including Parkinson’s disease (PD) and amyotrophic lateral sclerosis (ALS). SVA_67, a retrotransposon insertion polymorphism, is located in a 1.8 Mb region of high linkage disequilibrium, called the *MAPT* locus, which is known to contribute to increased risk of developing PD, frontotemporal dementia and other tauopathies. To investigate the role of SVA_67 in directing differential gene expression at this locus, we characterised the impact of SVA_67 allele dosage on isoform expression of several genes in the *MAPT* locus using the datasets from both the Parkinson’s Progression Markers Initiative and New York Genome Center Consortium Target ALS cohort. The Parkinson’s data was from gene expression in the blood and the ALS data from a variety of CNS regions and allowed us to demonstrate that SVA_67 presence or absence correlated with both isoform- and tissue-specific expression of multiple genes at this locus. This study highlights the importance of addressing SVA polymorphism in disease genetics to gain insight into a better understanding of the role of these regulatory domains to a variety of neurodegenerative diseases.

## Introduction

Neurodegenerative diseases, including Parkinson’s disease (PD) and amyotrophic lateral sclerosis (ALS), are complex disorders involving interaction of genetic and environmental factors (Emamzadeh and Surguchov, 2018). Genome-wide association studies (GWAS) and targeted single nucleotide polymorphism (SNP) studies have allowed the identification of many loci and genetic mutations and polymorphisms associated with these diseases (Taylor et al., 2016; van Rheenen et al., 2016; Nalls et al., 2019; Blauwendraat et al., 2020). However, further analyses are needed to characterise and identify the source of the current missing heritability of these diseases.

**FIGURE 1:**
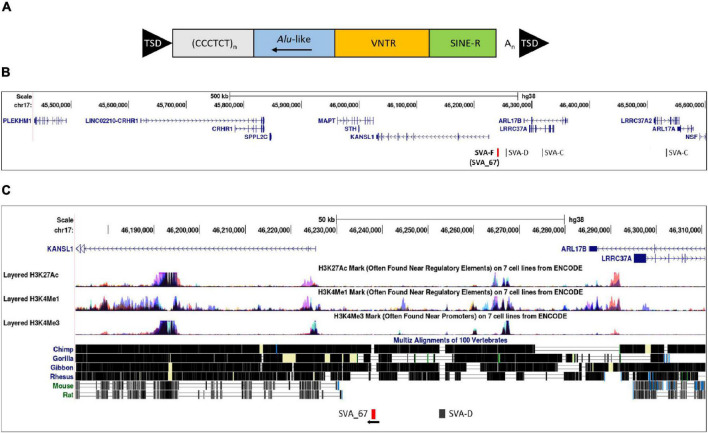
Structure of a full-length SVA and schematic representation of the location of SVA_67. **(A)** A typical SVA element (full-length) contains five domains. Considered 5′–3′, SVAs are composed of a (CCCTCT)_*n*_ hexamer repeat, an inverted *Alu*-like region, a GC-rich variable number tandem repeat (VNTR), a SINE-R domain and a poly A-tail. SVA elements are flanked by target site duplications (TSDs). **(B)** Schematic modified from UCSC genome browser hg38 of the *MAPT* locus on chromosome 17. This locus contains multiple genes and four SVAs including the SVA_67 RIP (red bar). Six genes (*MAPT*, *KANSL1*, *CRHR1*, *LRRC37A*, *PLEKHM1* and *ARL17A*) were selected for analysis. SVA_67 (chr17:46,237,519–46,238,226) is anti-sense with respect to the orientation (defined as their 5′ TSS) of the *LRRC37A*, *CRHR1* and *MAPT* gene, and shows a sense orientation relative to the *KANSL1*, *ARL17A* and *PLEKHM1* gene. **(C)** ENCODE data from UCSC showing the levels of enrichment of histone marks in proximity of SVA_67. Signals for H3K27Ac (marker of active regulatory domains associated with active enhancer elements), H3K4Me1 (marker of regulatory domains associated with enhancers) and H3K4Me3 (marker of a regulatory domains associated with promoters) are indicated. The region has been overlaid with a conservation track of several primates and rodents which shows that SVA_67 is human specific.

Non-coding repetitive DNA is often an overlooked source of genetic variation. Repetitive DNA can be found in both static and mobile forms, whereby transposable elements (TEs) belonging to the latter class are capable of mobilising throughout the genome (Bourque et al., 2018). Originally dismissed as “junk” DNA, TEs are now known to contribute to the regulation and evolution of the genome as well as to be involved in genetic instability and disease progression (Ayarpadikannan and Kim, 2014). Based on their transposition strategy and intermediates formed, TEs are split into two families named DNA transposons and retrotransposons (Savage et al., 2019). Retrotransposon sequences can propagate *via* a “copy-and-paste” mechanism leading to a new copy at a new genomic locus of the host genome (Elbarbary et al., 2016). SINE-VNTR-*Alu* (SVA) elements, approximately 0.7–4 kb in length, are one member of the non-LTR (long terminal repeat) retrotransposon family with 2,700–3,000 copies present in the reference human genome. They consist of a 5′ CT-rich hexamer domain (CT element), an antisense *Alu*-like region, a central GC-rich variable number tandem repeat (VNTR, each repeat typically 30–50 bp in length), a SINE-R domain and a 3′ poly A tail (Figure 1A; Hancks and Kazazian, 2010; Gianfrancesco et al., 2017). SVAs, classified A–F in order of evolutionary age based on their SINE region (Wang et al., 2005), are important contributors to genetic diversity by a variety of mechanisms which include acting as transcriptional regulatory domains and thus modulating gene expression profiles, for instance, by transcription factor (TF) binding or altering patterns of methylation (Hancks et al., 2011). Ongoing mobilisation of SVAs has led to insertions being polymorphic for their presence or absence and are thus named retrotransposon insertion polymorphisms (RIPs) (Hancks and Kazazian, 2016; Gianfrancesco et al., 2019). It should be noted that the evolutionarily youngest classes of SVAs (D-F1) may be of special interest for human physiology, health and evolution since they have introduced hominid-specific insertions which can exert novel regulatory potential to specific genomic loci (Vasieva et al., 2017).

To date, there are at least 13 disease-causing SVA insertions (Hancks and Kazazian, 2016; Bychkov et al., 2021) including the insertion causing X-linked dystonia parkinsonism (XDP), where an SVA-F element was found in intron 32 of the TATA-box binding protein associated factor 1 (*TAF1*) gene (Makino et al., 2007; Aneichyk et al., 2018; Delvallée et al., 2021; Yamamoto et al., 2021). Interestingly, this SVA insertion was not only found to be associated with reduced expression of *TAF1*, alternative splicing and intron retention, but also the length of its CT hexamer repeat inversely correlated with age of onset of the disease (Bragg et al., 2017). Furthermore, polymorphic SVA insertions have been shown to regulate gene expression or isoform expression *via* intron retention in a population specific manner (Makino et al., 2007; Wang et al., 2017). This highlights their ability to have an impact at many levels on genetic processing and contribute to phenotypic differences within a variety of diseases including neurodegenerative disorders such as PD or ALS. We recently characterised SVA RIPs utilising whole genome sequencing, transcriptomic and clinical data in the Parkinson’s Progression Markers Initiative (PPMI) cohort, which was designed to help understand PD aetiology, identify progression markers, and enhance development of novel therapeutics (Initiative, 2011; Pfaff et al., 2021). Eighty-one reference genome SVAs polymorphic for their presence/absence were identified, seven of which were linked with PD progression and with differential gene expression using whole blood RNA sequencing data (Pfaff et al., 2021). One of these RIPs, SVA_67, is located 12 kb upstream of the KAT8 regulatory NSL complex subunit 1 (*KANSL1*) gene which is part of the microtubule-associated protein tau (*MAPT*) locus (Figure 1B). The structurally complex *MAPT* locus contains a 900-kb inversion and is characterised by two predominant haplotypes (*H1* and *H2*) and the presence of SVA_67 is part of *H1* (direct inversion) while *H2* (inverted) is specified by its absence (Zody et al., 2008; Wider et al., 2010). This locus contains several genes including *MAPT* in which mutations can cause, or polymorphisms are correlated with the neurodegenerative diseases frontotemporal dementia (FTD) with parkinsonism and progressive supranuclear palsy (PSP) (Im et al., 2015; Strang et al., 2019). In relation to ALS, it has been shown that 12.5% of patients with behavioural-variant FTD develop ALS, and mild features of motor neuron involvement have been reported in about 40% of patients with FTD (Bang et al., 2015; van Es et al., 2017).

SVAs have the potential to exert regulatory influences on genes distant from the closest gene to which they are found. As non-coding RIPs may lead to interpersonal differences in expression patterns, we aimed to extend our previous findings (Pfaff et al., 2021) by analysing the association of the SVA_67 RIP with isoform expression of six genes located in the block at the *MAPT* locus including *MAPT*, *KANSL1*, corticotropin releasing hormone receptor 1 (*CRHR1*), leucine rich repeat containing 37A (*LRRC37A*), pleckstrin homology and RUN domain containing M1 (*PLEKHM1*) and ADP ribosylation factor like GTPase 17A (*ARL17A*). Using the datasets from both the PPMI and New York Genome Center Consortium Target ALS (henceforth NYGC ALS) cohort, we could show that the SVA_67 genotype was significantly associated with differential isoform expression of all genes of interest in the *MAPT* locus. Our data only addressed the ability of the SVA to alter isoform expression and current data does not give us sufficient power to address isoform expression in disease progression. These approaches could nevertheless lead to a more precise understanding of transposable elements as contributors to a variety of neurodegenerative diseases.

## Materials and Methods

### Bioinformatic Analysis of the *MAPT* Locus

The *MAPT* locus was examined using the UCSC Genome Browser hg38^1^, which contains a set of tools allowing the visualisation of a defined genomic region. The ‘‘Repeat Masker’’ tool^2^ was used to screen and identify low complexity DNA sequences and interspersed repeats, including retrotransposons and tandem repeat DNA within the reference genome. This method, where “Repeat Masker” annotations were overlaid with conservation data, specifically the “vertebrate multiz alignment” and conservation of 100 vertebrate species from “Phylogenetic Analysis with Space/Time” models (PHAST program) (Hubisz et al., 2011), allowed us to determine if the SVA of interest (SVA_67) was human specific or present in other primates. To analyse the potential of this non-coding region to have regulatory function, the genomic region of SVA_67 was overlaid with data from “The Encyclopaedia of DNA Elements” (ENCODE) (Consortium, 2012). Signals for H3K4Me1, H3K4Me3 and H3K27Ac histone marks, which are found near regulatory regions, promoters, and active regulatory elements, respectively, were analysed.

### Genotyping of SVA_67 in Parkinson’s Progression Markers Initiative and NYGC Amyotrophic Lateral Sclerosis Cohort and Analysis of Differential Isoform Expression

In this study, we utilised data from the Parkinson’s Progression Markers Initiative (PPMI) and New York Genome Center Consortium Target ALS (NYGC ALS) cohort. The structural variant caller Delly2^3^ (Rausch et al., 2012), with default settings, was used to genotype SVA_67 in individuals of the PPMI and the NYGC ALS cohorts (Pfaff et al., 2021). The PPMI is a longitudinal study and SVA_67 was genotyped in 608 individuals, including 179 healthy controls, 371 PD and 58 SWEDD (scans without evidence of dopaminergic deficit) subjects (Marek et al., 2018). For the NYGC cohort, the SVA_67 genotype was available for 197 subjects, including 142 ALS, 29 ALS + other neurological disorders (OND), 4 OND, 21 healthy controls and 1 other motor neuron disease (spinal bulbar muscular atrophy). OND diagnosis can include the following diseases: dementia with lewy bodies, peripheral neuropathy, Alzheimer’s disease, multiple system atrophy, frontotemporal dementia, argyrophilic grain disease, cerebral amyloid angiopathy, acute meningitis, cerebrovascular disease, chronic traumatic encephalopathy, and Parkinson’s disease. RNA-seq data from different tissues of these subjects from the NYGC ALS cohort were available. The majority represented tissues from the central nervous system (CNS), which were complemented by choroid and liver tissues. Taken together, RNA-seq data from the following tissues (total 1,004) were included: cerebellum (163), 141 frontal cortex (141), medial motor cortex (120), lateral motor cortex (119), unspecified motor cortex (10), occipital cortex (75), temporal cortex (5), sensory cortex (2), lumbar spinal cord (114), cervical spinal cord (132), thoracic spinal cord (72), choroid (32) and liver (19).

In order to evaluate the effect of SVA RIP genotypes on the expression profile, differential isoform expression analysis based on the PPMI whole blood RNA-seq data and NYGC ALS RNA-seq data was performed on all subjects. In this analysis, all subjects (cases and controls) were combined. Isoform quantification of RNA-seq data was performed by using the Salmon tool^4^. Salmon-generated quant files were imported into R using *tximport* function from the *tximport* package (Soneson et al., 2015) of R. Counts were extracted with the *DESeqDataSetFromTximport* function and raw counts were normalised using the median-of-ratios method, implemented in the *DESeq2* package (Love et al., 2014). The *DESeq2* package in R was also used to detect statistically significant differences in the isoform expression profiles between the different genotypes of SVA_67. The association of three different genotypes (*AA*, *PA*, *PP*) based on the presence (*P*) or absence (*A*) of SVA_67 was analysed. The *ggplot2* package (Wickham and Sievert, 2016) in R containing the *geom_boxplot* function was used to visualise the data specifying the *stat_summary* function to *mean*. Six genes (*MAPT*, *KANSL1*, *PLEKHM1*, *CRHR1*, *LRCC37A*, and *ARL17A*) centred around this RIP at the *MAPT* locus were selected for analysis. The unpaired Wilcoxon test was used to compare two independent groups of samples and to demonstrate statistical significance.

## Results

### Bioinformatic Analysis of the *MAPT* Locus

The *MAPT* locus on chromosome 17q21.31 was analysed using UCSC genome browser, specifically evaluating SVAs, ENCODE data and evolutionary DNA conservation over this region (Figure 1). Using the “Repeat Masker” data track for analysis, four SVA retrotransposons were identified, including two SVA-C elements as well as one element of the classes D and F (Figure 1B). UCSC genome browser additionally indicated three potential SVA-A elements which were present in genome version GRCh38/hg38 but were not identified in the previous version GRCh37/hg19. Upon inspection of the primary sequence of these elements we excluded these elements with a length of 58, 127 and 128 bp, respectively, because their sequences did not align with characteristic structures (e.g., CT element, VNTR or 3′ poly A) of SVA elements. These sequences align with sequences of Alu elements and were therefore incorrectly annotated as SVA elements. We previously identified the SVA-F element, termed SVA_67, as a RIP showing that it transposed relatively recently in evolutionarily age. SVA_67 (hg38, chr17:46,237,519–46,238,226), located 12 kb upstream of the *KANSL1* gene, represented a truncated element with a length of 707 bp. This element was anti-sense with respect to the orientation of the genes [defined by their 5′ transcriptional start sites (TSS)] *LRRC37A*, *CRHR1* and *MAPT*, and showed a sense orientation relative to *KANSL1*, *ARL17A* and *PLEKHM1* (Figures 1B,C). These six genes were selected for isoform expression analyses. The distances between SVA_67 and the TSS of analysed gene isoforms (Table 1) ranged from approximately 12–800,000 kb and are summarised in Table 1. Using the “vertebrate multiz alignment” and conservation track of 100 vertebrate species, this analysis showed that this genomic region was not conserved in chimps, gorillas, gibbons, rhesus macaques, rats, and mice, indicating that SVA_67 was human specific (Figure 1C). We overlaid the genomic region of SVA_67 with data from ENCODE to analyse if this SVA was associated with signals for H3K4Me1 (marker of regulatory domains associated with enhancers), H3K4Me3 (marker of regulatory domains associated with promoters) and H3K27Ac (marker of active regulatory domains associated with active enhancer elements) marks (Figure 1C). No major histone marks associated with active chromatin were observed in proximity to SVA_67, although this may not be surprising as SVAs are not well captured and aligned to specific genomic locations utilising short read DNA sequence due to the repetitive nature and primary sequence homology of the SVA retrotransposons.

**TABLE 1:** Analysed gene isoforms in this study and their specifics.

Name	Transcript ID	bp	Protein	Biotype	Location on chromosome 17	Distance SVA_67 to TSS (bp)
MAPT-201	ENST00000262410	6,815	833aa	PC	45,894,554–46,028,334	342,965
MAPT-202	ENST00000334239	1,107	352aa	PC	45,894,668–46,024,183	342,851
MAPT-203	ENST00000344290	2,609	736aa	PC	45,894,560–46,024,425	342,959
MAPT-204	ENST00000351559	5,639	441aa	PC	45,894,554–46,028,334	342,965
MAPT-205	ENST00000415613	2,331	776aa	PC	45,962,338–46,024,171	275,181
MAPT-206	ENST00000420682	1,239	412aa	PC	45,962,338–46,024,171	275,181
MAPT-207	ENST00000431008	1,233	410aa	PC	45,962,338–46,024,171	275,181
MAPT-208	ENST00000446361	5,345	383aa	PC	45,894,674–46,028,334	342,845
MAPT-209	ENST00000535772	1,353	381aa	PC	45,894,551–46,024,225	342,968
MAPT-210	ENST00000570299	889	-	PT	45,894,576–46,018,730	342,943
MAPT-211	ENST00000571311	544	59aa	NMD	45,894,566–45,978,425	342,953
MAPT-212	ENST00000571987	2,277	758aa	PC	45,962,338–46,024,171	275,181
MAPT-213	ENST00000572440	5,015	-	RI	45,975,492–45,980,506	262,027
MAPT-215	ENST00000576518	6,778	-	RI	45,972,783–46,024,431	264,736
MAPT-217	ENST00000680542	2,988	412aa	PC	45,894,663–46,025,873	342,856
MAPT-218	ENST00000680674	1,936	424aa	PC	45,894,527–46,024,655	342,992
KANSL1-201	ENST00000262419	5,309	1105aa	PC	46,029,956–46,192,800	44,719
KANSL1-202	ENST00000432791	5,574	1105aa	PC	46,029,916–46,193,429	44,090
KANSL1-203	ENST00000571698	2,276	678aa	PC	46,039,870–46,193,901	43,618
KANSL1-204	ENST00000572218	9,095	-	RI	46,029,916–46,046,121	191,398
KANSL1-205	ENST00000572904	4,973	1105aa	PC	46,029,916–46,223,676	13,843
KANSL1-206	ENST00000573286	5,437	-	RI	46,037,325–46,043,101	194,418
KANSL1-207	ENST00000573682	358	-	RI	46,032,165–46,033,512	204,007
KANSL1-208	ENST00000574590	5,147	1104aa	PC	46,029,916–46,225,389	12,130
KANSL1-209	ENST00000574655	1,681	-	PT	46,152,904–46,225,371	12,148
KANSL1-210	ENST00000574963	1,493	-	RI	46,031,074–46,032,566	204,953
KANSL1-211	ENST00000575318	4,935	1041aa	PC	46,029,917–46,225,367	12,152
KANSL1-212	ENST00000576137	815	-	RI	46,033,099–46,039,075	198,444
KANSL1-213	ENST00000576248	452	-	PT	46,119,967–46,171,223	66,296
KANSL1-214	ENST00000576739	592	9aa	PC	46,172,117–46,224,902	12,617
KANSL1-215	ENST00000576870	2,848	-	RI	46,029,918–46,040,065	197,454
KANSL1-216	ENST00000577114	629	-	PT	46,050,650–46,121,338	116,181
KANSL1-217	ENST00000638269	3,365	-	RI	46,092,870–46,225,361	12,158
KANSL1-218	ENST00000638275	5,352	1041aa	PC	46,029,917–46,193,400	44,119
KANSL1-220	ENST00000638551	899	-	PT	46,032,186–46,035,160	202,359
KANSL1-221	ENST00000638902	1,971	-	RI	46,170,277–46,223,685	13,834
KANSL1-222	ENST00000639099	1,724	-	PT	46,152,904–46,225,367	12,152
KANSL1-223	ENST00000639150	1,815	523aa	PC	46,033,080–46,224,693	12,826
KANSL1-224	ENST00000639279	387	-	PT	46,088,428–46,094,641	142,878
KANSL1-225	ENST00000639356	1,717	-	PT	46,148,125–46,225,367	12,152
KANSL1-226	ENST00000639375	3,392	-	RI	46,049,399–46,225,355	12,164
KANSL1-228	ENST00000639520	2,655	-	PT	46,221,186–46,225,376	12,143
KANSL1-229	ENST00000639531	2,731	897aa	PC	46,032,257–46,172,183	65,336
KANSL1-231	ENST00000639853	1,425	475aa	PC	46,038,636–46,171,416	66,103
KANSL1-233	ENST00000640519	1,970	-	PT	46,033,045–46,035,924	201,595
KANSL1-236	ENST00000648792	4,933	1061aa	PC	46,029,985–46,225,373	12,146
PLEKHM1-201	ENST00000430334	5,240	1056aa	PC	45,435,900–45,490,721	746,798
PLEKHM1-202	ENST00000446609	2,825	63aa	NMD	45,440,163–45,487,858	749,661
PLEKHM1-203	ENST00000579131	499	-	PT	45,437,916–45,441,059	796,460
PLEKHM1-204	ENST00000579197	4,995	63aa	NMD	45,435,904–45,490,728	746,791
PLEKHM1-205	ENST00000580205	2,385	-	PT	45,452,938–45,460,432	777,087
PLEKHM1-206	ENST00000580404	2,628	-	PT	45,435,903–45,446,273	791,246
PLEKHM1-207	ENST00000581448	3,292	335aa	NMD	45,437,471–45,490,729	746,790
PLEKHM1-208	ENST00000581932	1,163	-	RI	45,477,074–45,482,525	754,994
PLEKHM1-209	ENST00000582035	1,834	-	RI	45,452,938–45,454,771	782,748
PLEKHM1-211	ENST00000583150	479	-	PT	45,439,615–45,448,112	789,407
PLEKHM1-212	ENST00000584420	603	140aa	PC	45,475,211–45,490,738	746,781
PLEKHM1-213	ENST00000585506	332	-	RI	45,439,514–45,440,373	797,146
PLEKHM1-214	ENST00000586084	553	99aa	NMD	45,475,696–45,490,741	746,778
PLEKHM1-215	ENST00000586562	550	-	PT	45,475,597–45,490,749	746,770
PLEKHM1-216	ENST00000589780	587	152aa	PC	45,475,366–45,490,741	746,778
PLEKHM1-217	ENST00000590991	327	45aa	NMD	45,439,546–45,445,507	792,012
PLEKHM1-218	ENST00000591580	650	113aa	PC	45,437,549–45,445,533	791,986
CRHR1-201	ENST00000293493	2,621	430aa	PC	45,784,280–45,835,827	453,239
CRHR1-202	ENST00000314537	2,537	415aa	PC	45,784,320–45,835,828	453,199
CRHR1-203	ENST00000339069	2,490	314aa	PC	45,784,280–45,835,827	453,239
CRHR1-204	ENST00000347197	2,462	145aa	NMD	45,784,307–45,835,825	453,212
CRHR1-205	ENST00000352855	1,146	375aa	PC	45,784,527–45,834,764	452,992
CRHR1-206	ENST00000398285	2,399	444aa	PC	45,784,545–45,835,828	452,974
CRHR1-207	ENST00000535778	1,272	52aa	NMD	45,833,138–45,835,644	404,381
CRHR1-208	ENST00000577353	1,206	401aa	PC	45,784,545–45,834,764	452,974
CRHR1-209	ENST00000580876	223	75aa	PC	45,833,138–45,834,645	404,381
CRHR1-210	ENST00000580955	479	93aa	NMD	45,807,074–45,830,125	430,445
CRHR1-211	ENST00000581479	606	-	RI	45,830,400–45,833,202	407,119
CRHR1-212	ENST00000582766	828	-	RI	45,784,569–45,830,940	452,950
CRHR1-213	ENST00000583888	777	152aa	NMD	45,816,558–45,834,841	420,961
CRHR1-214	ENST00000619154	2,370	154aa	PC	45,784,280–45,835,827	453,239
ARL17A-202	ENST00000336125	5,242	177aa	PC	46,552,756–46,579,691	341,465
ARL17A-203	ENST00000445552	798	125aa	PC	46,516,702–46,579,670	342,151
ARL17A-206	ENST00000622488	524	88aa	PC	46,528,685–46,579,673	342,154
LRRC37A-201	ENST00000320254	5,177	1700aa	PC	46,295,131–46,337,794	56,905
LRRC37A-202	ENST00000393465	4,997	1635aa	PC	46,295,099–46,337,777	57,580
LRRC37A-203	ENST00000496930	3,860	738aa	PC	46,292,733–46,337,792	55,214

*PC, protein coding; PT, processed transcript; NMD, non-sense mediated decay; RI, retained intron; TSS, transcriptional start site.*

### Genotype of SVA_67 Is Significantly Associated With Differential Isoform Expression of Several Genes in the *MAPT* Locus

**FIGURE 2:**
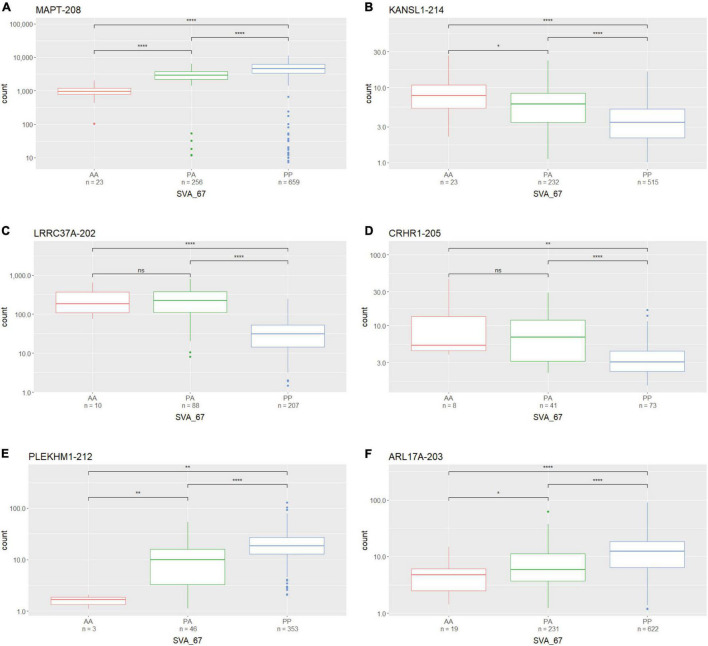
The expression of several gene isoforms at the *MAPT* locus is associated with SVA_67 genotype. Association of SVA_67 genotype with expression of isoform MAPT-208 **(A)**, KANSL1-214 **(B)**, LRRC37A-202 **(C)**, CRHR1-205 **(D)**, PLEKHM1-212 **(E)** and ARL17A-203 **(F)** was analysed by the presence or absence of the SVA RIP (AA, PA, PP) using the NYGC ALS cohort. Wilcoxon test was applied to demonstrate statistical significance indicated as asterisks. *P ≤ 0.05, **P ≤ 0.01, ***P ≤ 0.001, ****P ≤ 0.0001, ns > 0.05.

We assessed whether the SVA_67 genotype correlated with isoform expression of six genes (*MAPT*, *KANSL1*, *PLEKHM1*, *CRHR1*, *LRCC37A*, *ARL17A*) centred around this RIP at the *MAPT* locus (Figure 1B). Transcriptomic data of the PPMI and NYGC ALS cohort was utilised to determine if the SVA RIP had an influence on isoform expression. The association of three different genotypes (*AA*, *PA*, *PP*) based on the presence (*P*) or absence (*A*) of SVA_67 was analysed. Initial analysis of one isoform of each gene of interest using the NYGC ALS cohort demonstrated that SVA_67 allele dosage is significantly associated with differential expression of the specific isoforms MAPT-208, KANSL1-214, LRRC37A-202, CRHR1-205, PLEKHM1-212 and ARL17A-203 (Figure 2). Three of these isoforms (MAPT-208, PLEKHM1-212 and ARL17A-203) had significantly increased expression with *PP* genotype compared to both *PA* and *AA* genotype (Figures 2A,E,F). This was in contrast with the levels of expression of the isoforms KANSL1-214, LRRC37A-202 and CRHR1-205 where the opposite pattern was observed (Figures 2B–D). When extending the analysis for all detectable isoforms of the genes of interest using the NYGC ALS cohort, 14/16 *MAPT*, 3/3 *LRRC37A*, 3/3 *ARL17A*, 7/12 *CRHR1*, 12/17 *PLEKHM1* and 23/28 *KANSL1* isoforms were significantly associated with at least one SVA_67 genotype (Table 2 and Supplementary Figures 1, 3–5, 7, 9). When utilising the PPMI cohort, 2/4 *MAPT*, 3/3 *LRRC37A*, 2/2 *ARL17A*, 5/6 *CRHR1*, 7/9 *PLEKHM1* and 7/7 *KANSL1* isoforms were significantly associated with at least one SVA_67 genotype (Table 2 and Supplementary Figures 2–4, 6, 8, 10). More isoforms of these genes were detected in the NYGC ALS RNAseq than blood derived from the PPMI cohort. The majority of the corresponding RNAseq data derived from tissues of the CNS (brain and spinal cord) with just a small portion from choroid and liver. Further characterisation and validation will be required to address the significance of that data; however, it could be as simple as the heterogeneity of the CNS compared to blood. Analysed isoforms of each gene and additional information including isoform ID and biotype are summarised in Table 1.

**TABLE 2:** Summary of the effect of SVA_67 allele dosage on isoform expression of genes in the *MAPT* locus.

Gene	Analysed isoforms	Significant association of SVA_67 allele dosage on isoform expression		
		NYGC ALS cohort	PPMI cohort	Tissue specific influence on ≥ 1 isoform?
*ARL17A*	3	3/3	2/2	Yes
*CRHR1*	14	7/12	5/6	Yes
*KANSL1*	30	23/28	7/7	Yes
*LRRC37A*	3	3/3	3/3	Yes
*MAPT*	16	14/16	2/4	No
*PLEKHM1*	17	12/17	7/9	Yes

### Presence of SVA_67 Correlates With Gene Expression in an Isoform-Specific Manner

**FIGURE 3:**
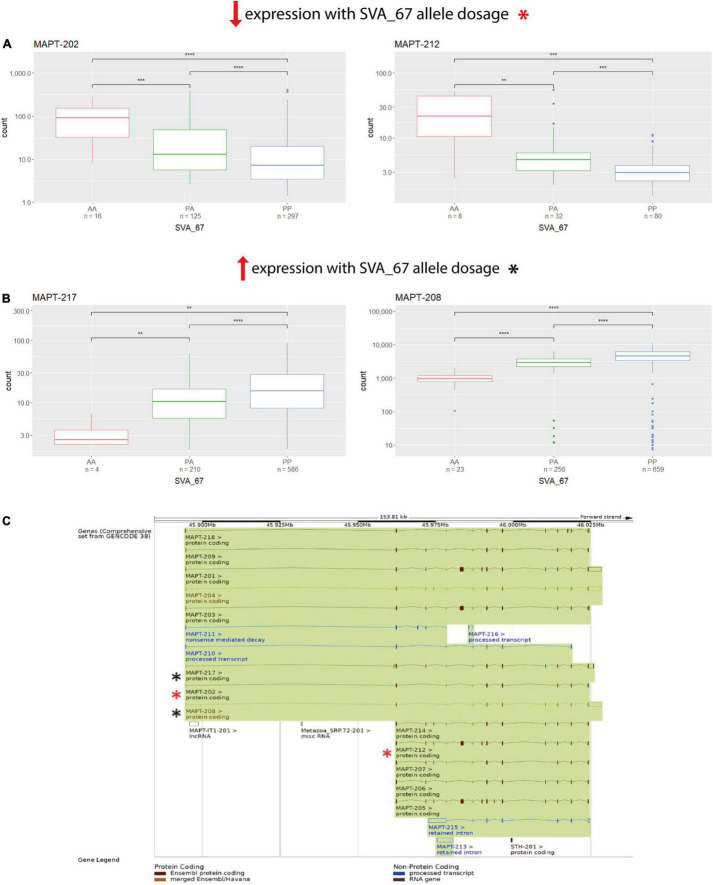
SVA_67 influences expression in an isoform-specific manner. Using the NYGC ALS cohort, isoforms MAPT-202 and MAPT-212 show significantly reduced expression with PP genotype **(A)**, while the opposite effect is observed for isoforms MAPT-217 and MAPT-208 **(B)**. Wilcoxon test was applied to demonstrate statistical significance indicated as asterisks. *P ≤ 0.05, **P ≤ 0.01, ***P ≤ 0.001, ****P ≤ 0.0001, ns > 0.05. **(C)** Overview of the *MAPT* gene and all its isoforms as shown on Ensembl. Boxes represent exons, connecting lines introns. Filled boxes show coding sequence, and empty boxes UTRs (untranslated regions). Analysed isoforms of interest in panels **(A,B)** are highlighted as asterisks (red/black).

We next assessed if SVA_67 had the same observed influence on each isoform of a gene regarding positive or negative impact on expression. A major consideration when analysing this data was that the expression could be from different cell types in the brain and therefore such positive or negative findings could be generated by two different cell populations. Nevertheless, utilising the NYGC ALS cohort, there was at least one isoform of each analysed gene present that demonstrated higher and lower expression, respectively, in individuals with *PP* genotypes compared to both *PA* and *AA* genotypes. For example, when addressing *MAPT*, the isoforms MAPT-202 and MAPT-212 showed significantly reduced expression with *PP* genotype compared to both *PA* and *AA* (Figure 3A), while two other isoforms of this same gene (MAPT-208 and MAPT-217) showed the opposite effect (Figure 3B). All four displayed isoforms represented protein coding isoforms encoding distinct proteins with different lengths and there was no association with a specific TSS although several are defined for the gene (Figure 3C and Table 1). This isoform-specific effect of SVA_67 on expression was also visible when using the PPMI cohort, where subjects with *PP* genotypes showed significantly increased and reduced, respectively, expression of a specific isoform compared to *AA*, *PA* or both *PA* and *AA* genotypes. For example, *KANSL1* isoforms KANSL1-201 and KANSL1-211 showed significantly increased expression with *PP* genotype compared to both *PA* and *AA*, while isoforms KANSL1-209 and KANSL1-214 were associated with reduced expression when the subjects had both alleles of SVA_67 present (Supplementary Figure 10). No statistical significance regarding positive and negative effect was obtained for *MAPT* isoforms in the PPMI cohort. Here, subjects with PP genotype correlated significantly with increased expression of isoform MAPT-208 (*PP* vs *PA* and *AA*) and MAPT-215 (*PP* vs *AA*) (Supplementary Figure 2). The association of SVA_67 genotype did not reach statistical significance for the isoform MAPT-211, however, a trend was visible which indicated a reduced expression with *PP* and *PA* genotypes compared to subjects having no allele of SVA_67 present.

### A Tissue Specific Influence of SVA_67 Allele Dosage on Isoform Expression

**FIGURE 4:**
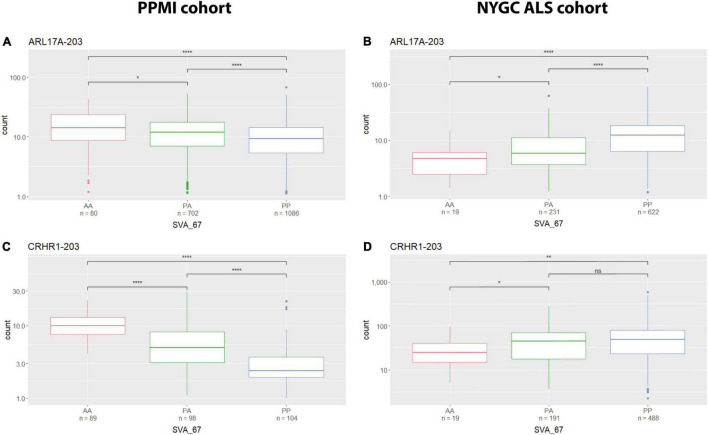
Tissue specific influence of SVA_67 genotype. Using the PPMI cohort, isoforms ARL17A-203 **(A)** and CRHR1-203 **(C)** show significantly reduced expression with PP genotype, while the opposite effect is observed when using the NYGC ALS cohort **(B,D)**. Wilcoxon test was applied to demonstrate statistical significance indicated as asterisks. *P ≤ 0.05, **P ≤ 0.01, ***P ≤ 0.001, ****P ≤ 0.0001, ns > 0.05.

We analysed the effect of SVA_67 on specific isoforms in different tissues by comparing the influence of this SVA RIP in two different datasets (PPMI and NYGC ALS cohort). The six isoforms LRRC37A-201, ARL17A-203, CRHR1-203, PLEKHM1-202, KANSL1-208 and KANSL1-209 were differentially influenced by SVA_67 allele dosage demonstrating opposing effects on expression when comparing both datasets (Table 2 and Supplementary Figures 3, 7–10). Using the PPMI cohort, isoform PLEKHM1-202 showed significantly increased expression with *PP* genotype compared to both *PA* and *AA* genotype, while the opposite effect is visible when utilising the NYGC ALS cohort. We do not believe this is disease specific as we have both cases and controls in our datasets to give us power to address transcriptional changes, however, isoform association with disease can be considered in the future as the number of cases and controls increases in such data sets. A tissue-specific effect was also detectable for the two *KANSL1* isoforms KANSL1-208 and KANSL1-209. When using the PPMI dataset for analysis, both isoforms were associated with reduced expression when having two alleles of SVA_67 present, while the same genotype led to a decreased expression when using the NYGC ALS cohort for analysis. Regarding LRRC37A-201, this isoform showed an increased expression with one copy of SVA_67 present compared to *AA* in the PPMI cohort. Again, the opposite effect was visible when utilising the NYGC ALS dataset. ARL17A-203 and CRHR1-203 isoforms showed a significantly reduced expression with *PP* genotype compared to both *PA* and *AA* genotype (PPMI dataset) (Figures 4A,C), while the opposite effect was visible when utilising the NYGC ALS cohort (Figures 4B,D).

## Discussion

In this study we analysed the impact of SVA_67 allele dosage on isoform expression of six genes (*MAPT*, *KANSL1*, *CRHR1*, *PLEKHM1*, *ARL17A* and *LRRC37A*) at the *MAPT* locus (Figure 1) in order to gain insight into the role of SVAs to modify the transcriptome. Using two different datasets (PPMI and NYGC ALS cohort) for analysis, we could demonstrate that SVA_67 was significantly associated with 1) changes in expression of multiple genes at this locus and 2) both differential isoform and tissue specific expression of these genes (Figures 2–4 and Table 2). The data is consistent with SVAs having functional consequences for gene expression over long distances directly by activator or repressor mechanisms or their ability to modulate genome structure by looping mechanisms affecting 3D structures in such as transcriptional hubs (Ferrari et al., 2021).

SVAs have previously been demonstrated to be involved in genetic instability and disease progression *via* genome regulation mechanisms (Hancks and Kazazian, 2010, 2016; Ayarpadikannan and Kim, 2014). It has been demonstrated that SVAs can modulate gene expression profiles by various mechanisms, for instance, by transcription factor binding, altering patterns of methylation and interaction with distant promoters through 3D chromatin structure by recruiting CCCTC-binding factor (CTCF), a master regulator of 3D chromatin structure (Hancks et al., 2011; Wang et al., 2019). Indeed, TEs have shown to have multiple binding sites for CTCF which has a well-established role in chromatin looping and topologically associated domain (TAD) formation (Bourque et al., 2008; Schwalie et al., 2013; Kentepozidou et al., 2020; Pugacheva et al., 2020). This could be one model to allow SVA_67 to act at many genes to enhance or reduce gene isoform expression or alter chromatin structure *via* looping. This is consistent with the obtained results showing that SVA_67 was capable of affecting isoform expression at both the most proximal (e.g., *KANSL1*) and most distant (e.g., *PLEKHM1*) TSS relative to its location at the *MAPT* locus (Figure 2 and Supplementary Figures 7–10).

We have previously characterised the function of SVAs to modulate gene expression profiles by *in vitro* and *in vivo* models including an SVA upstream of the FUS RNA binding protein (*FUS*) gene in which genetic mutations have been linked to many diseases including ALS (Savage et al., 2014) and an SVA upstream of the Parkinsonism associated deglycase DJ-1 gene, also termed *PARK7*, a gene associated with PD (Savage et al., 2013). These demonstrated the action of SVAs to have classical regulatory domains when analysed in reporter gene constructs and indeed that the SVAs were a composite regulatory domain containing multiple functional domains. In our recent study, we could identify 81 reference SVAs polymorphic for their presence/absence, seven of which, including SVA_67, were associated with the progression of PD using the PPMI cohort (Pfaff et al., 2021). We have extended that previous work by addressing differential gene expression on an isoform-based level using the same cohort. We observed that SVA_67 had an isoform-specific correlation with gene expression showing potentially the capacity of SVA_67 to differentially modulate the transcriptome (Figure 3). Although we do not wish to draw conclusions regarding specific expression and disease progression, we do want to highlight the plethora of transcriptomic changes associated with one SVA insertion. We are not able to demonstrate that causation of differential gene expression is directed by the SVA which would await functional validation by such as CRISPR. No study to date has shown this regulatory influence of an SVA on an isoform-specific level, although intron retention has been proposed for the action of an SVA within an intron of the *TAF1* gene (Makino et al., 2007). However, intron retention is not operating at the *MAPT* locus as the SVA is intergenic in nature (Figure 1). To date, studies have focused predominantly on differential gene expression analyses, however, these approaches are limited as they do not account for isoform diversity (Makino et al., 2007; Hancks and Kazazian, 2010; Gianfrancesco et al., 2017; Hall et al., 2020). Many of the genes at this locus have been associated with CNS functional parameters (Caillet-Boudin et al., 2015; McEwan et al., 2015; Moreno-Igoa et al., 2015; de la Tremblaye et al., 2017) and encode more than one isoform which are generated, for instance, through mechanisms such as alternative splicing or alternative usage of transcription start sites (Elkon et al., 2013). It is important to differentiate between isoforms, as some of these can represent protein-coding isoforms with different functions and/or subcellular localisations, while others do not lead to a protein product (Table 1). Dick et al. (2020) have reported the first genome-wide study of differential transcript usage (DTU), the measure of the relative contribution of one isoform to overall gene expression, in PD. The authors demonstrated that PD subjects showed a decrease in the relative usage of a thioesterase superfamily member 5 (*THEM5*) transcript (involved in mitochondrial fatty acid metabolism), while the concomitant increase of a shorter isoform, which more likely localises to the extracellular space than to the mitochondria, may therefore not recapitulate the function of the full-length protein (Zhuravleva et al., 2012; Dick et al., 2020). Indeed, these observed changes in the relative expression of specific gene isoforms may affect the ratio of the resulting protein isoforms, which in turn could affect cellular signalling pathway or metabolism through variation of, for instance, function and subcellular localisation (Dick et al., 2020). In our study SVA_67 allele dosage was significantly associated, for instance, with *PLEKHM1* isoform expression (Figure 2 and Supplementary Figures 7, 8). McEwan et al. (2015) demonstrated in their study that PLEKHM1 regulates clearance of protein aggregates in an autophagy- and LC3-interacting region-dependent manner. Mutant or misfolded protein accumulation is implicated in the pathogenesis of multiple neurodegenerative diseases, and dysfunction or depletion of PLEKHM1 links this important regulator of protein aggregate removal to an increased risk of PD and ALS. Our data suggest that SVA_67 could influence *PLEKHM1* function by modulating isoform expression which could result in a gene specific DTU. This may have an impact on the potential role of *PLEKHM1* in maintaining appropriate cellular functions or even cell survival. Future work could address the role of *PLEKHM1* and other genes of interest in this study in neurodegenerative diseases by analysing the influence of isoform switches and DTU. More importantly it demonstrates the potential for multigenic regulatory effects of a single variant over a large region of the genome giving greater insight into the complex mechanisms that have to be factored in to understand how a variant is affecting not only genomic regulation but disease progression.

It is known that TE derived sequences can contribute to the regulation of the human genome and that domains with regulatory potential can function in a tissue-specific manner. We previously utilised Genotype-Tissue Expression (GTEx) database (Consortium, 2013) to characterise the potential of SVA_67 to act in other tissues using its proxy SNP (rs55653937) (Pfaff et al., 2021). This approach led, based on the findings that the tagging SNP for SVA_67 was identified as eQTL (expression quantitative trait loci) for over 30 genes including the six genes of interest in this study, to the assumption that SVA_67 could also influence gene expression in other tissues in addition to whole blood. We validated that by using transcriptomic data derived from different tissues (NYGC ALS vs PPMI) and demonstrated that SVA_67 correlated with a tissue-specific effect on isoform expression of six genes in the *MAPT* locus (Figure 4).

Our study presented here demonstrated that SVA insertions have the potential to influence expression of multiple genes over large distances and that regulation can be isoform specific. SVAs could be influencing the disease course of PD and ALS through modulation of isoform expression and usage which ultimately could affect protein levels and biological processes. Therefore, this study highlighted an additional type of variation to be considered at the *MAPT* locus and an added layer of complexity when analysing the missing heritability of neurodegenerative diseases.

## Data Availability Statement

Publicly available datasets were analysed in this study. This data can be found here: Raw data are available from the PPMI (www.ppmi-info.org/data) and the NYGC Target ALS (https://www.targetals.org/research/resources-for-scientists/) website.

## Author Contributions

AF, AP, VB, SK, and JQ contributed to study concept, design, analysis and interpretation of data, and critical revision of the manuscript for important intellectual content. AF drafted the manuscript. All authors reviewed and approved the final manuscript.

## Conflict of Interest

The authors declare that the research was conducted in the absence of any commercial or financial relationships that could be construed as a potential conflict of interest.

## Publisher’s Note

All claims expressed in this article are solely those of the authors and do not necessarily represent those of their affiliated organizations, or those of the publisher, the editors and the reviewers. Any product that may be evaluated in this article, or claim that may be made by its manufacturer, is not guaranteed or endorsed by the publisher.

## Footnotes

1
https://genome.ucsc.edu/

2
http://repeatmasker.org

3
https://github.com/dellytools/delly

4
https://salmon.readthedocs.io

## Abbreviations

ALS: amyotrophic lateral sclerosis
ARL17A: ADP ribosylation factor like GTPase 17A
CRHR1: corticotropin releasing hormone receptor 1
CTCF: CCCTC-binding factor
DTU: differential transcript usage
ENCODE: The Encyclopaedia of DNA Elements
eQTL: expression quantitative trait loci
FTD: frontotemporal dementia
FUS: FUS RNA binding protein
GTEx: Genotype-Tissue Expression
KANSL1: KAT8 regulatory NSL complex subunit 1
LRRC37A: leucine rich repeat containing 37A
MAPT: microtubule-associated protein tau
NYGC ALS: New York Genome Center Consortium Target ALS
PD: Parkinson’s disease
PLEKHM1: pleckstrin homology and RUN domain containing M1
PPMI: Parkinson’s Progression Marker Initiative
PSP: progressive supranuclear palsy
RIP: retrotransposon insertion polymorphism
SVA: SINE-VNTR-*Alu*
TAD: topologically associated domain
TAF1: TATA-box binding protein associated factor 1
TE: transposable element
THEM5: thioesterase superfamily member 5
TSS: transcriptional start site
VNTR: variable number tandem repeat
XDP: X-linked dystonia parkinsonism.
