# Supplementary material for: Characterisation of the Function of a SINE-VNTR-Alu Retrotransposon to Modulate Isoform Expression at the MAPT Locus
Source: Front Mol Neurosci. 2022 Mar 9;15:815695. doi: 10.3389/fnmol.2022.815695 (PMC8965460; doi:10.3389/fnmol.2022.815695)
Supplement: Supplementary file 1 [file Data_Sheet_1.PDF]

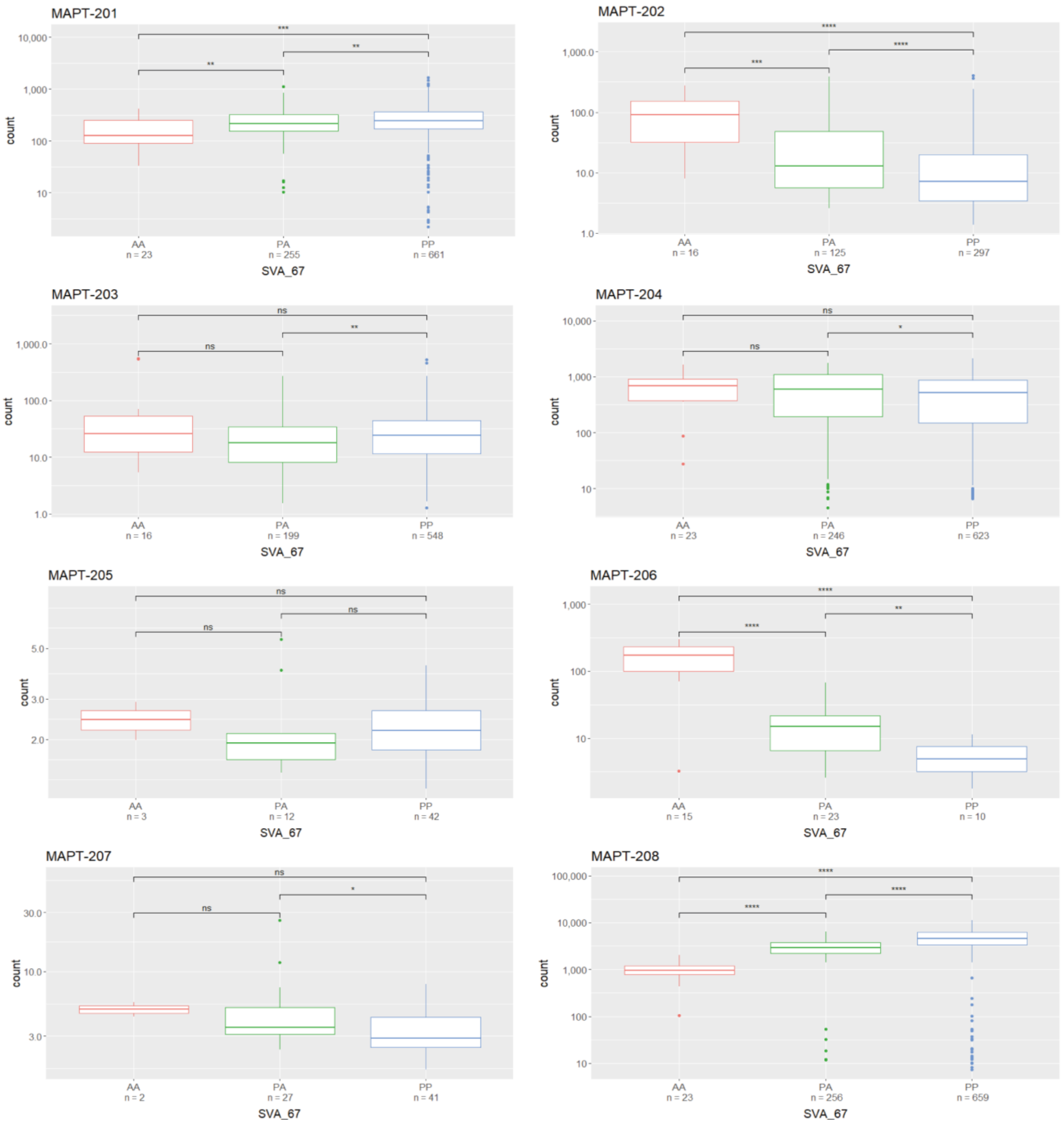

Supplementary Figure 1. Caption next page.

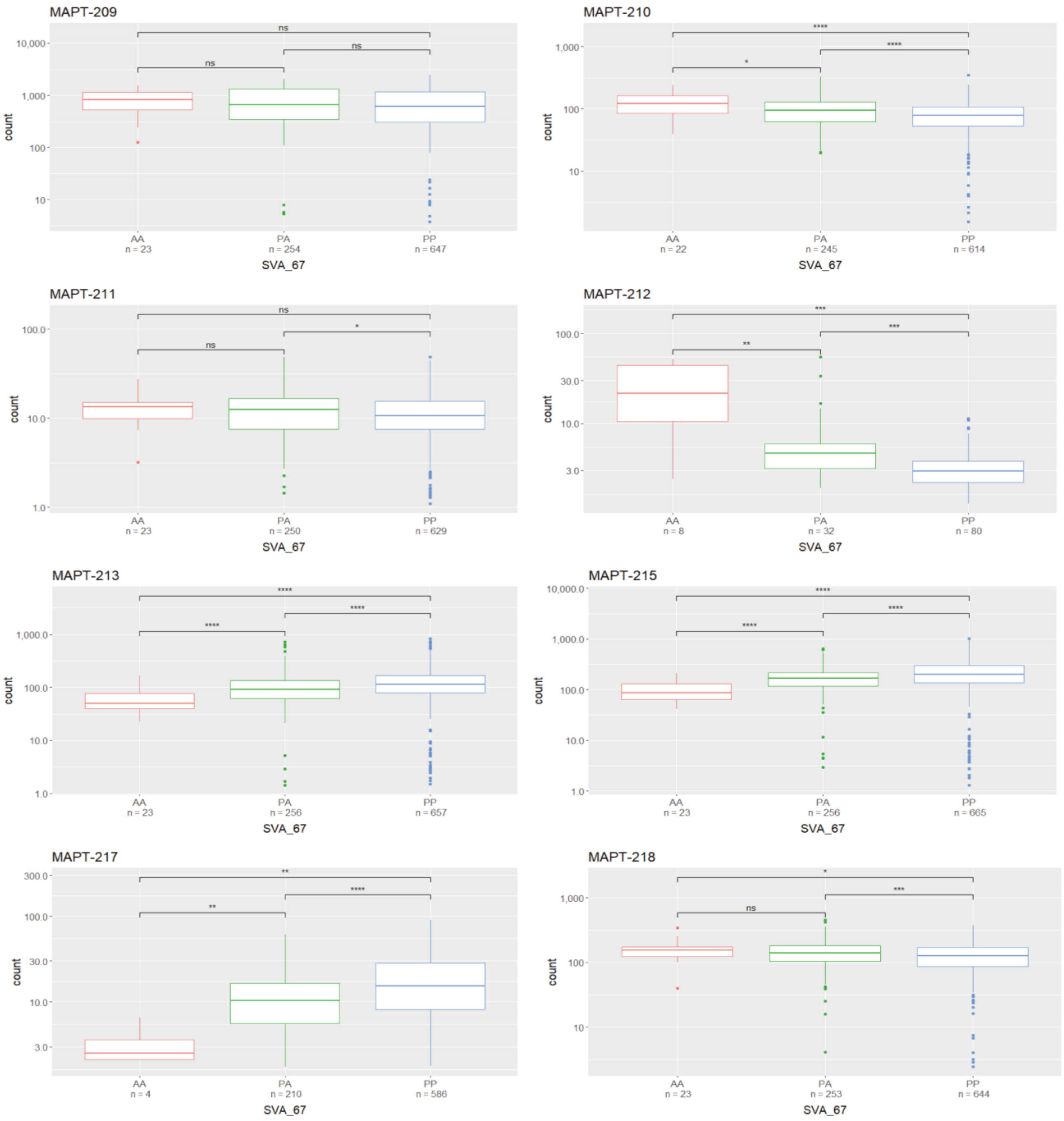

Supplementary Figure 2. Association of SVA_67 genotype with expression of *MAPT* isoforms using the NYGC ALS cohort. Three different genotypes (*AA*, *PA*, *PP*) were analysed. Wilcoxon test was used to demonstrate statistical significance indicated as asterisks. *P <= 0.05, **P <= 0.01, ***P <= 0.001, ****P <= 0.0001, ns > 0.05.

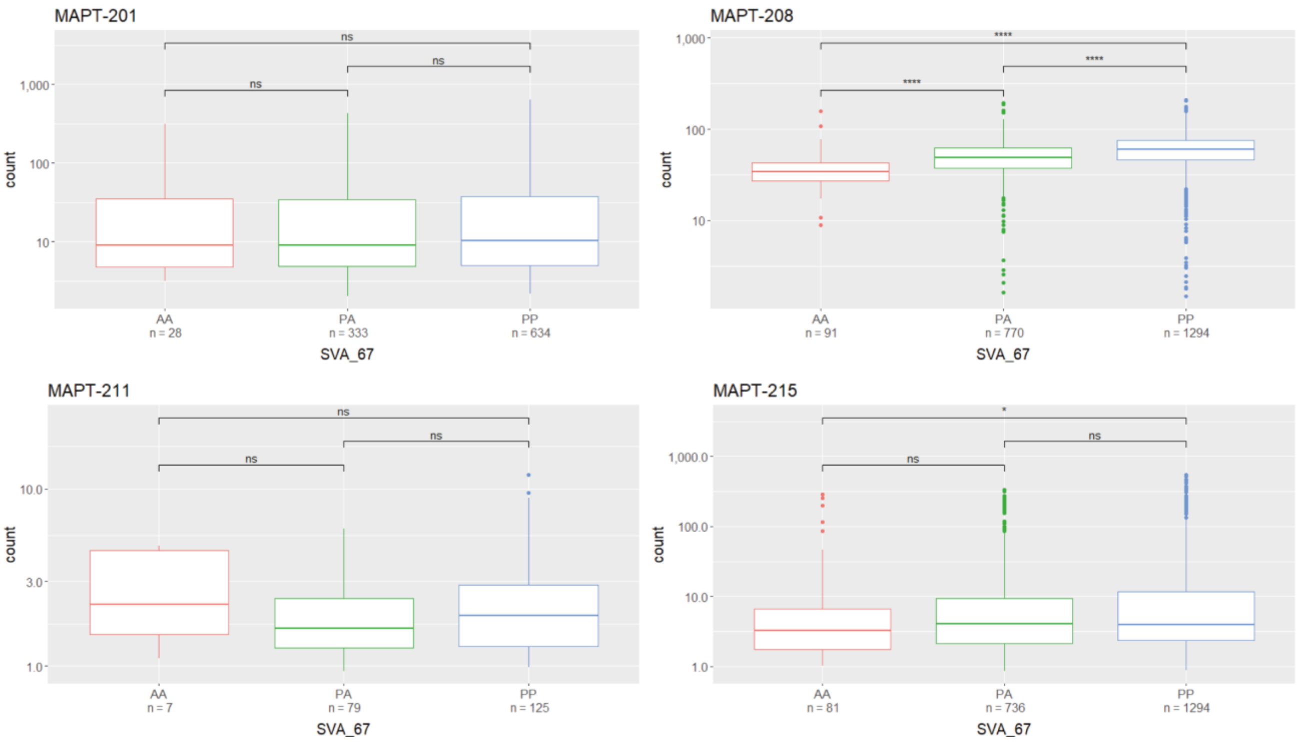

Supplementary Figure 2. Association of SVA_67 genotype with expression of *MAPT* isoforms using the PPMI cohort. Three different genotypes (*AA*, *PA*, *PP*) were analysed. Wilcoxon test was used to demonstrate statistical significance indicated as asterisks. *P <= 0.05, **P <= 0.01, ***P <= 0.001, ****P <= 0.0001, ns > 0.05.

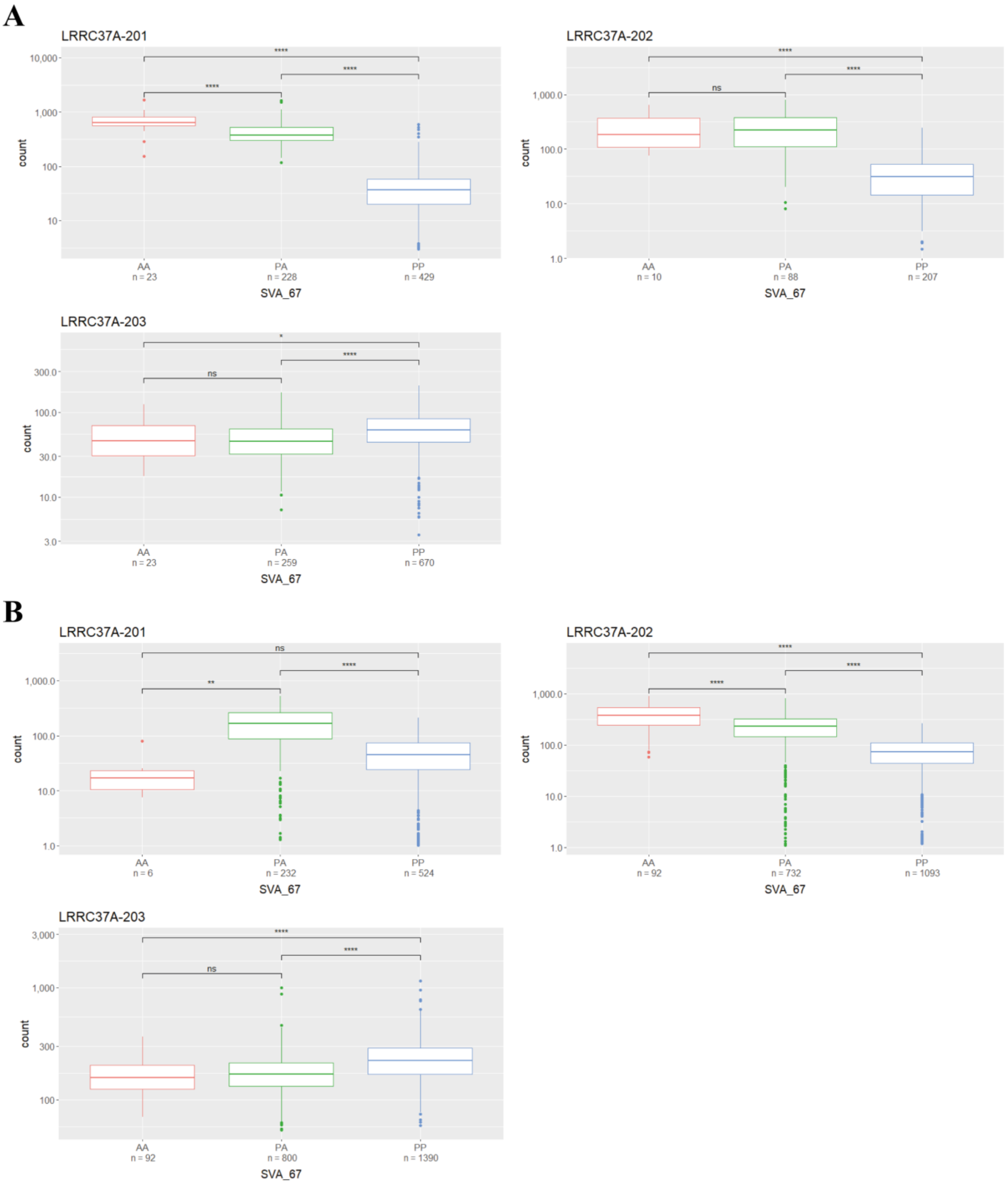

Supplementary Figure 3. Association of SVA_67 genotype with expression of *LRRC37A* isoforms using the NYGC ALS **(A)** and PPMI **(B)** cohort. Three different genotypes (*AA*, *PA*, *PP*) were analysed. Wilcoxon test was used to demonstrate statistical significance indicated as asterisks. *P <= 0.05, **P <= 0.01, ***P <= 0.001, ****P <= 0.0001, ns > 0.05.

A

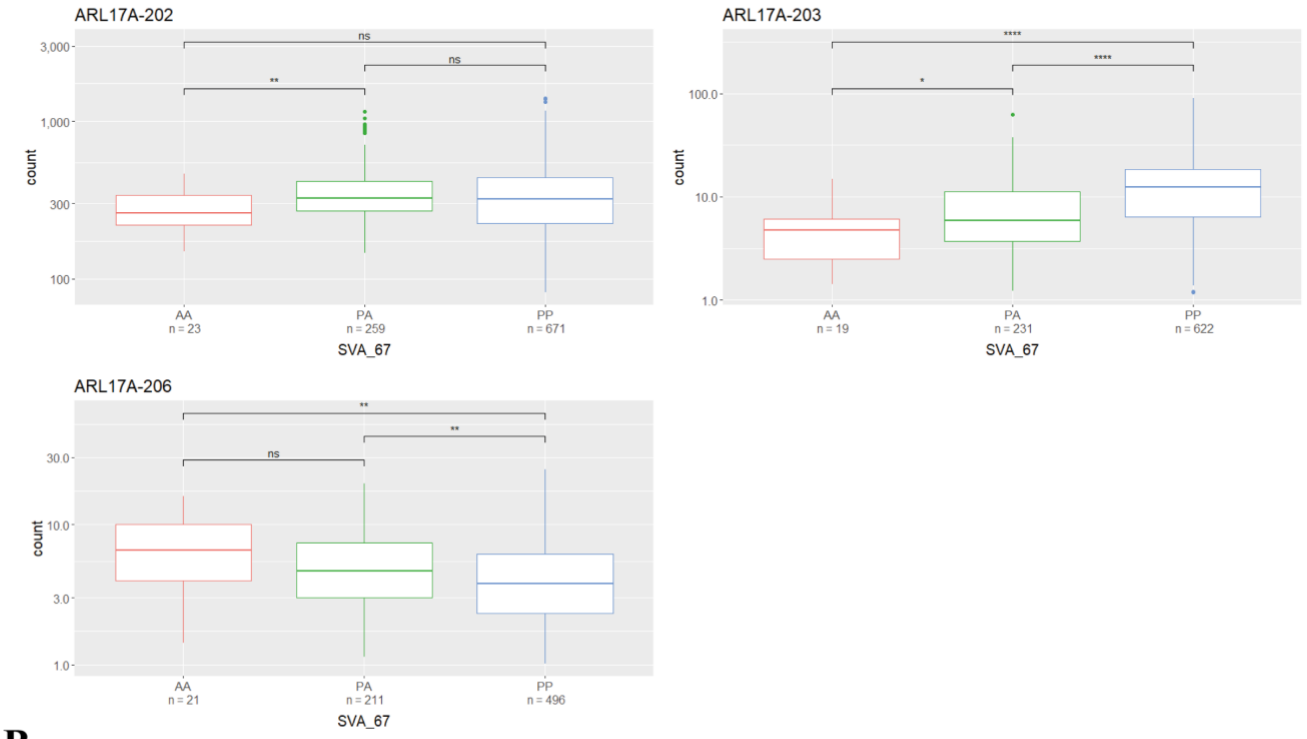

B

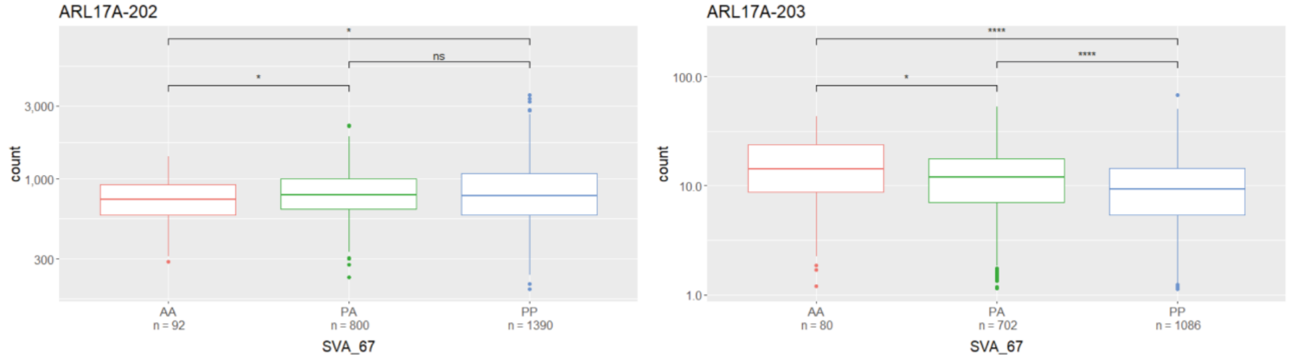

Supplementary Figure 4. Association of SVA_67 genotype with expression of *ARL17A* isoforms using the NYGC ALS **(A)** and PPMI **(B)** cohort. Three different genotypes (*AA*, *PA*, *PP*) were analysed. Wilcoxon test was used to demonstrate statistical significance indicated as asterisks. *P <= 0.05, **P <= 0.01, ***P <= 0.001, ****P <= 0.0001, ns > 0.05.

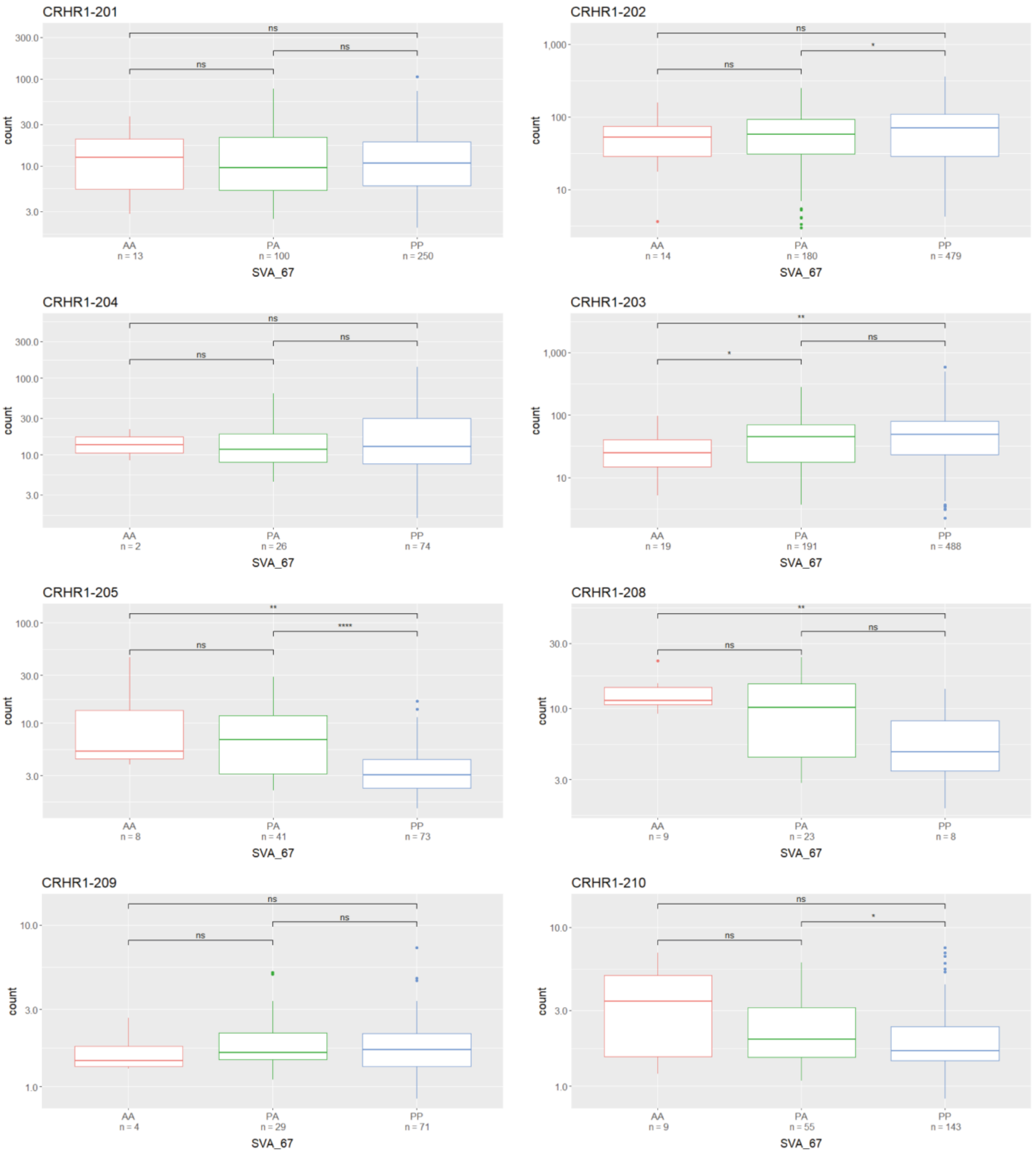

Supplementary Figure 5. Caption next page.

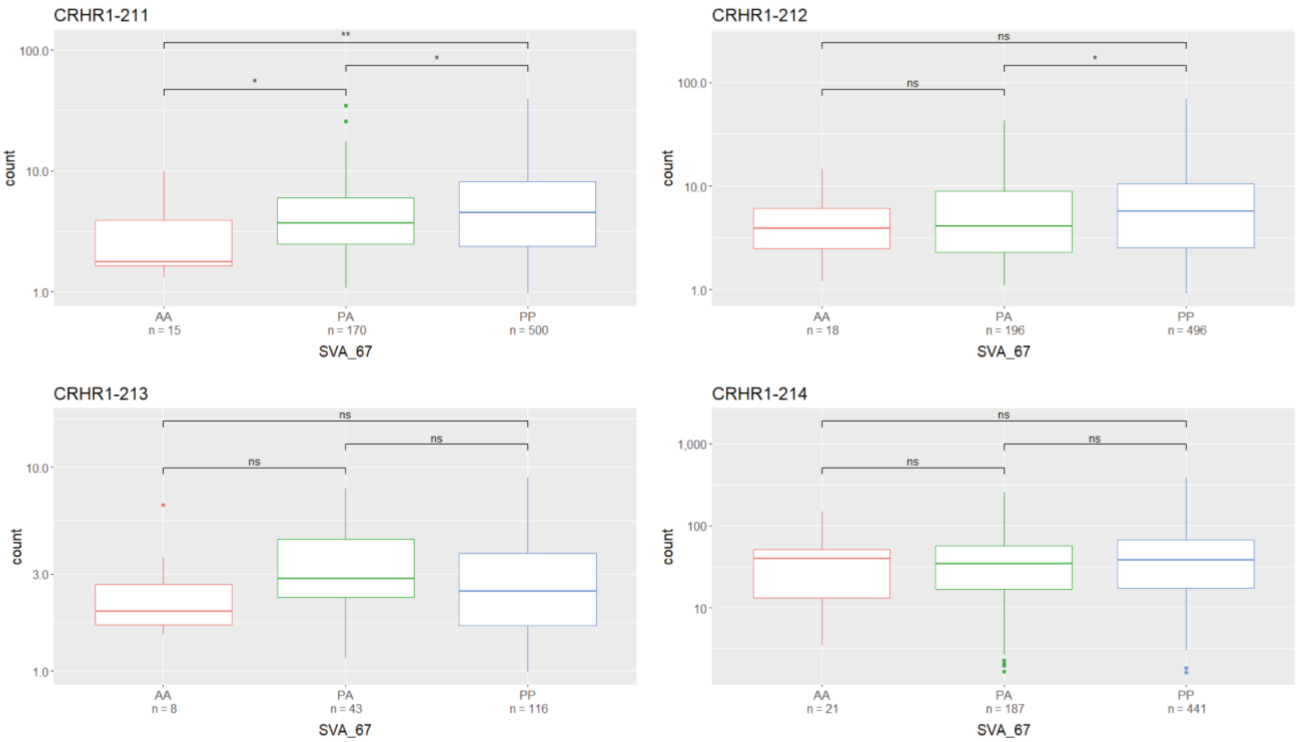

Supplementary Figure 5. Association of SVA_67 genotype with expression of *CRHR1* isoforms using the NYGC ALS cohort. Three different genotypes (*AA*, *PA*, *PP*) were analysed. Wilcoxon test was used to demonstrate statistical significance indicated as asterisks. *P <= 0.05, **P <= 0.01, ***P <= 0.001, ****P <= 0.0001, ns > 0.05.

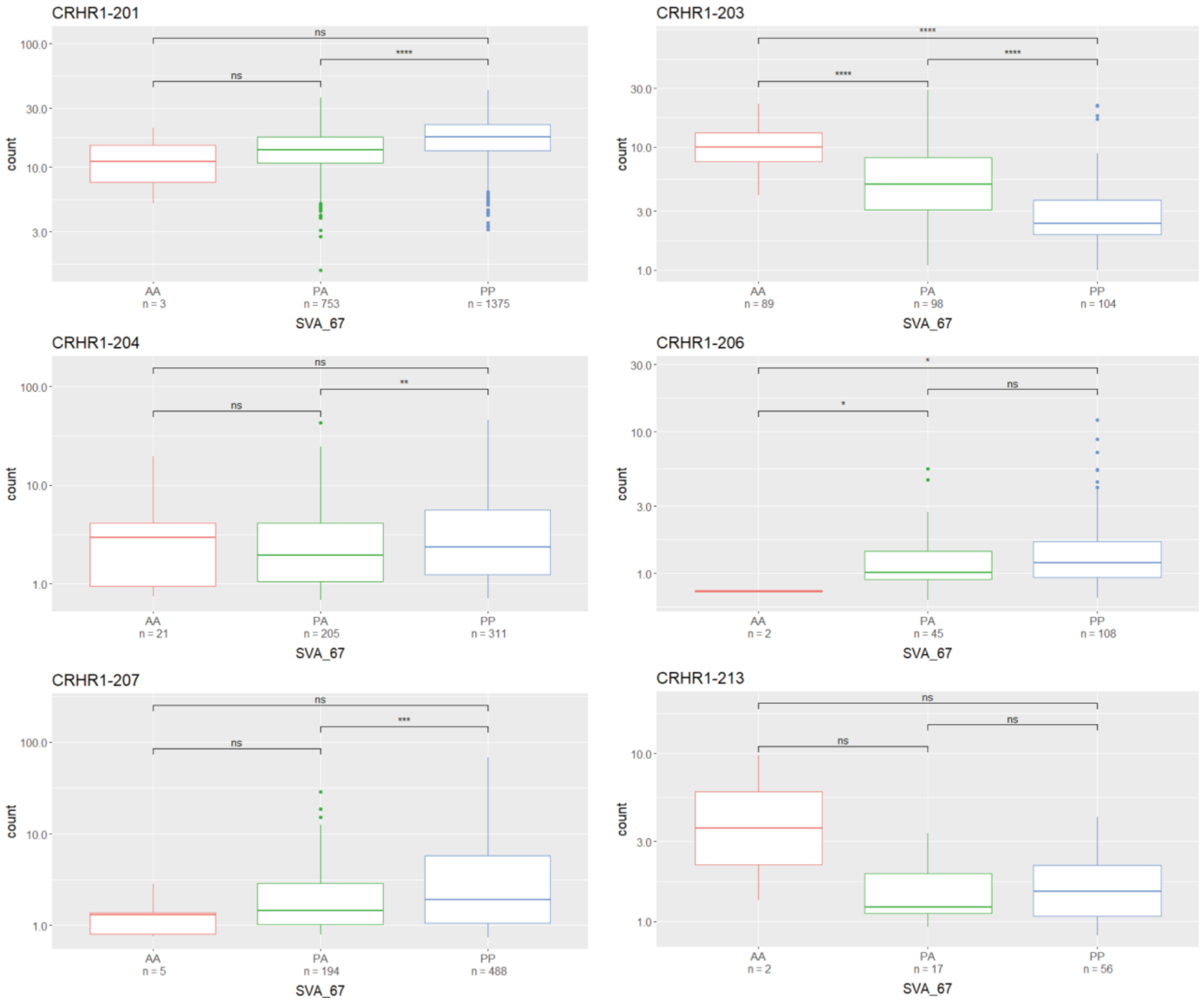

Supplementary Figure 6. Association of SVA_67 genotype with expression of *CRHR1* isoforms using the PPMI cohort. Three different genotypes (*AA*, *PA*, *PP*) were analysed. Wilcoxon test was used to demonstrate statistical significance indicated as asterisks. *P <= 0.05, **P <= 0.01, ***P <= 0.001, ****P <= 0.0001, ns > 0.05.

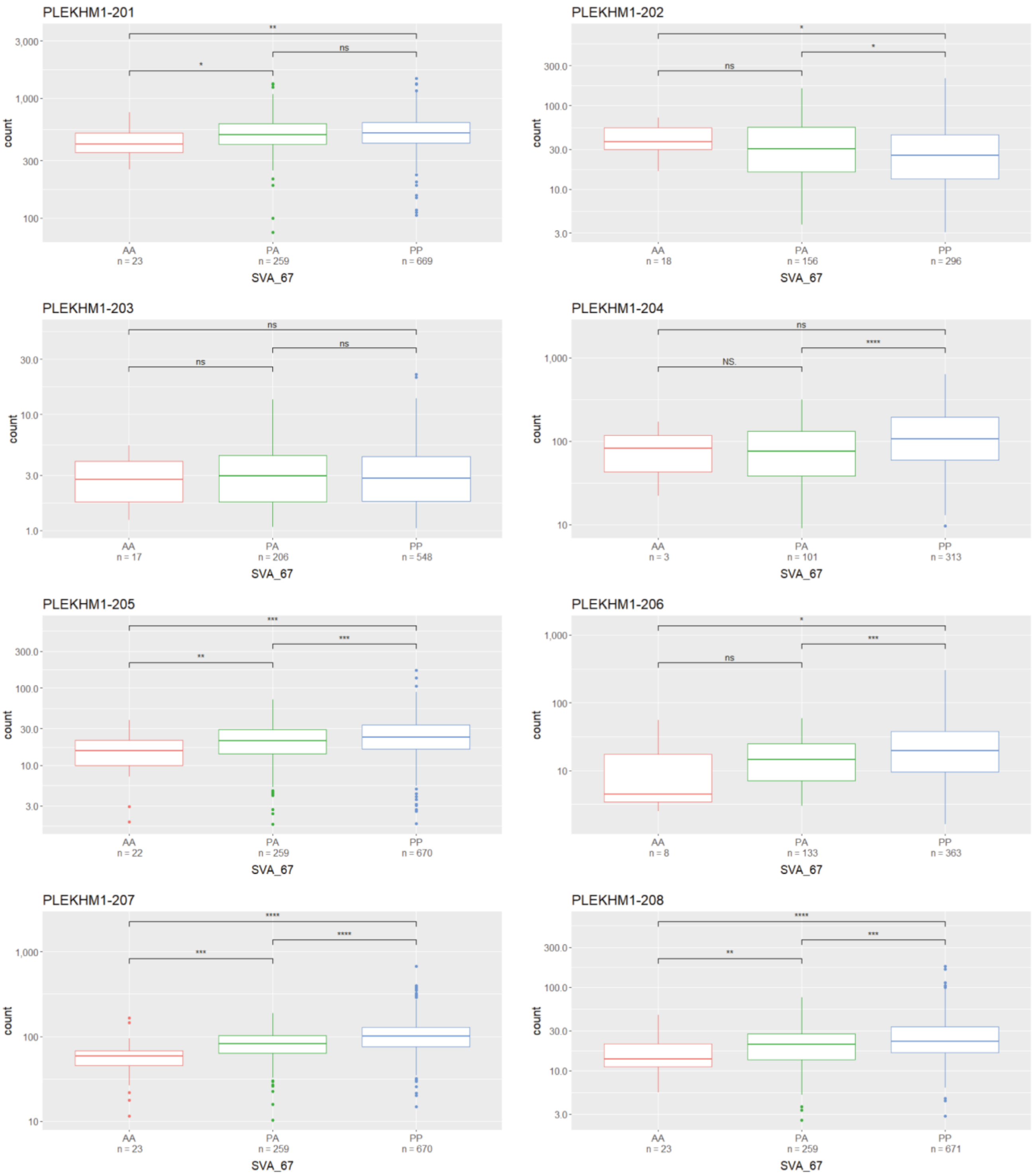

Supplementary Figure 7. Caption page 11.

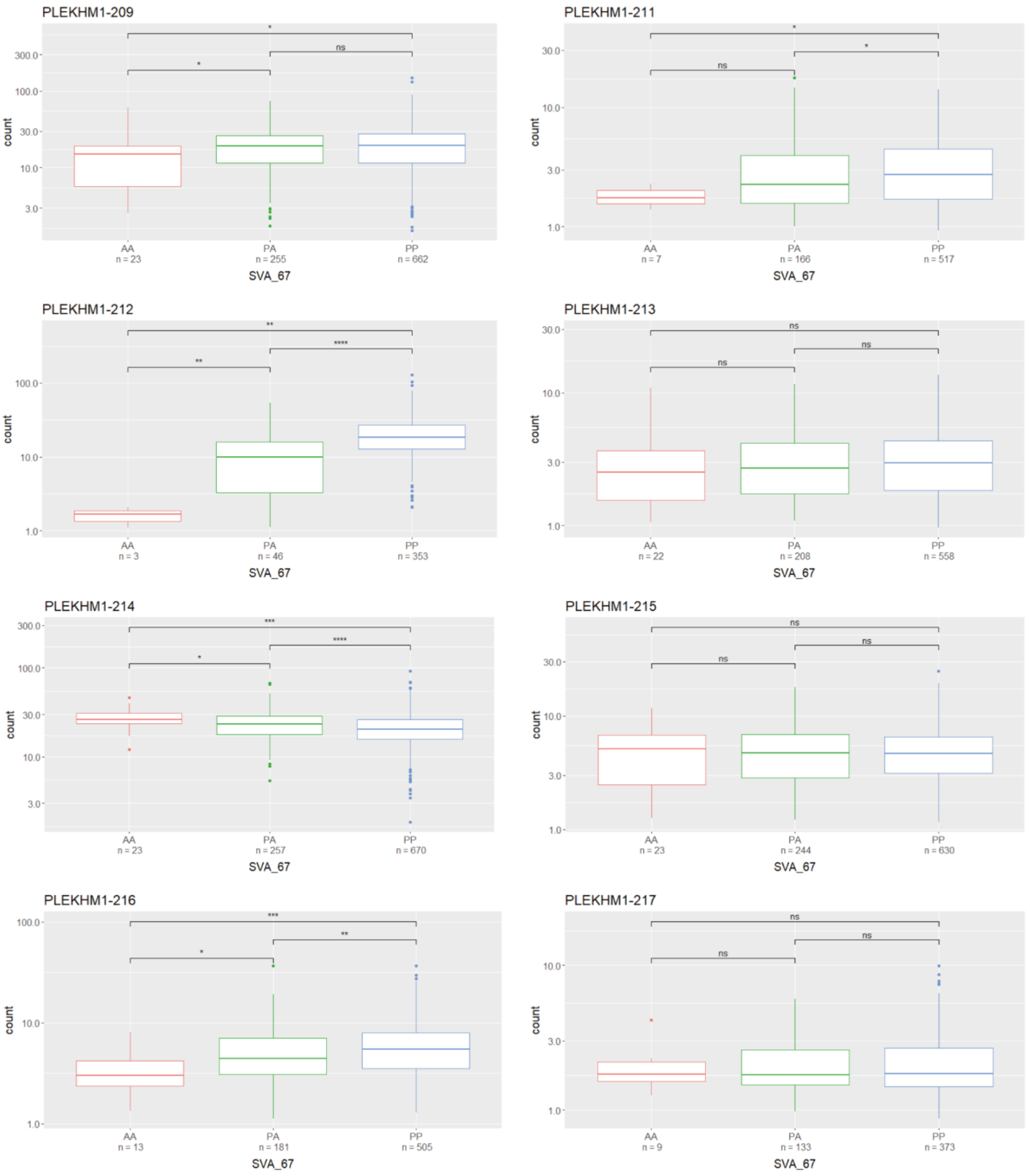

Supplementary Figure 7. Caption next page.

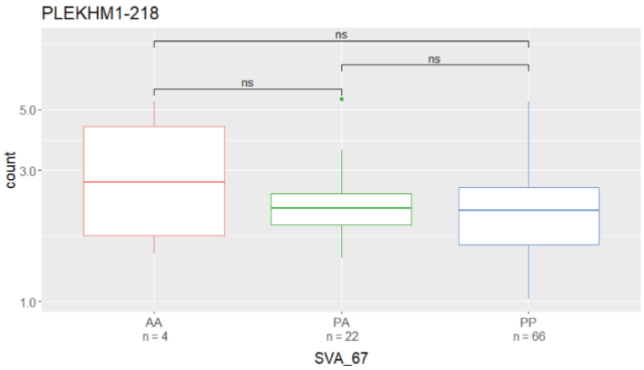

Supplementary Figure 7. Association of SVA_67 genotype with expression of *PLEKHM1* isoforms using the NYGC ALS cohort. Three different genotypes (*AA*, *PA*, *PP*) were analysed. Wilcoxon test was used to demonstrate statistical significance indicated as asterisks. *P <= 0.05, **P <= 0.01, ***P <= 0.001, ****P <= 0.0001, ns > 0.05.

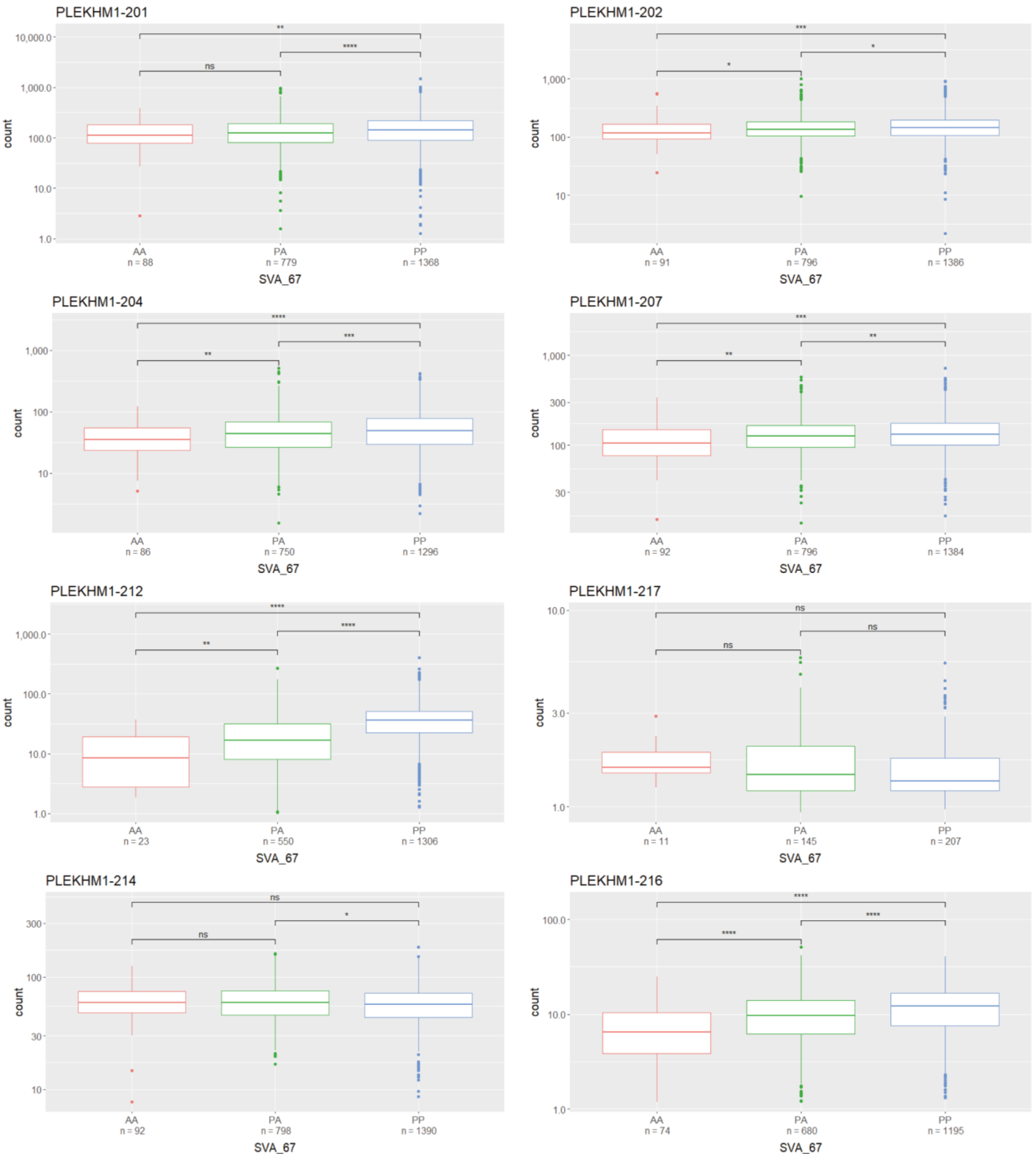

Supplementary Figure 8. Caption next page.

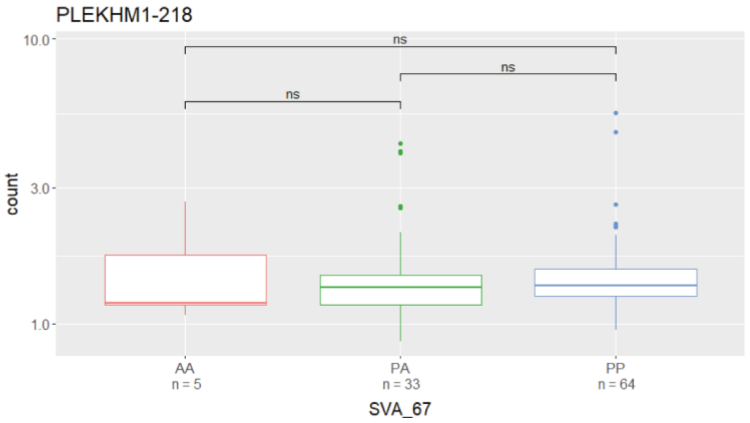

Supplementary Figure 8. Association of SVA_67 genotype with expression of *PLEKHM1* isoforms using the PPMI cohort. Three different genotypes (*AA*, *PA*, *PP*) were analysed. Wilcoxon test was used to demonstrate statistical significance indicated as asterisks. *P <= 0.05, **P <= 0.01, ***P <= 0.001, ****P <= 0.0001, ns > 0.05.

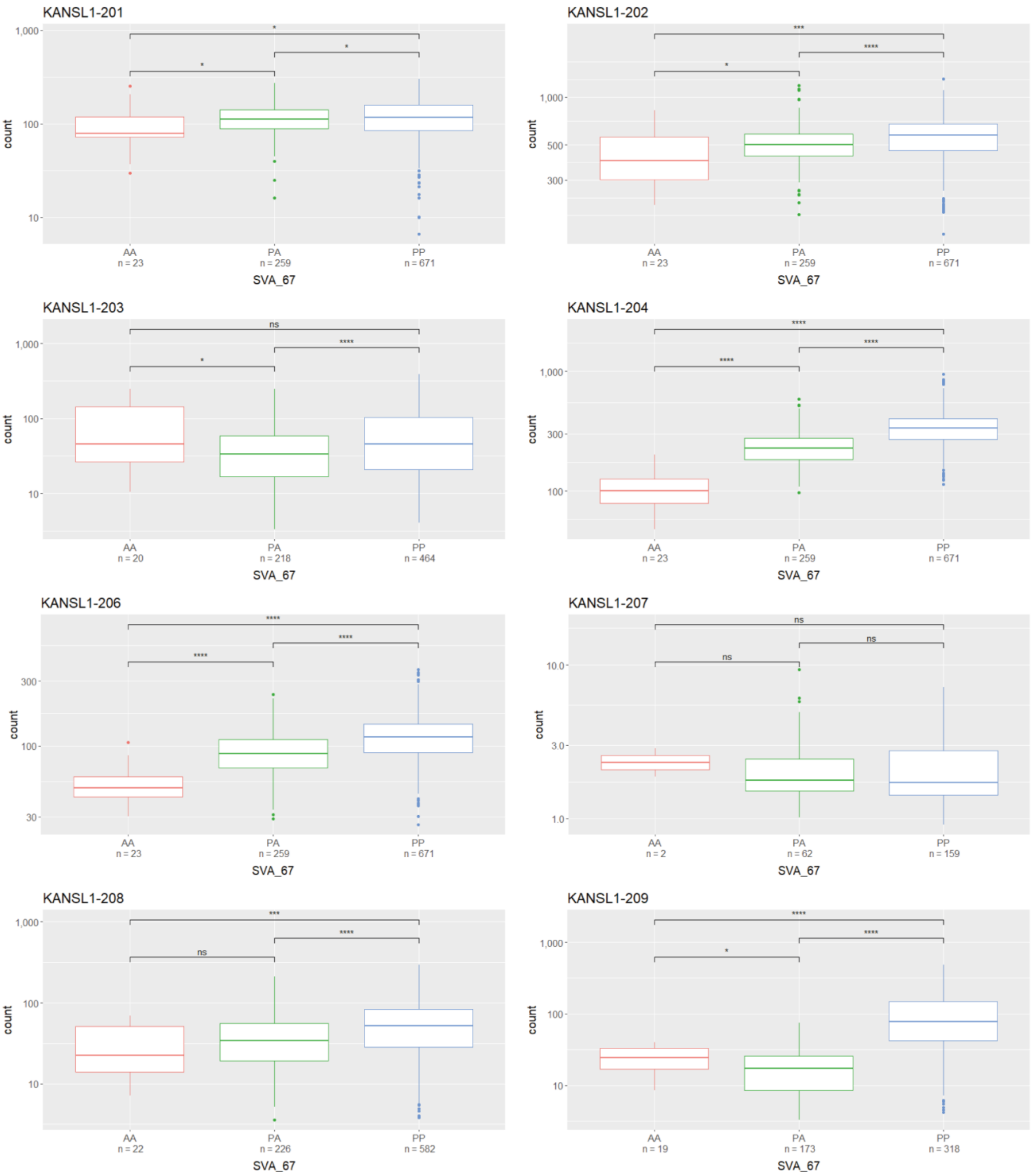

Supplementary Figure 9. Caption page 17..

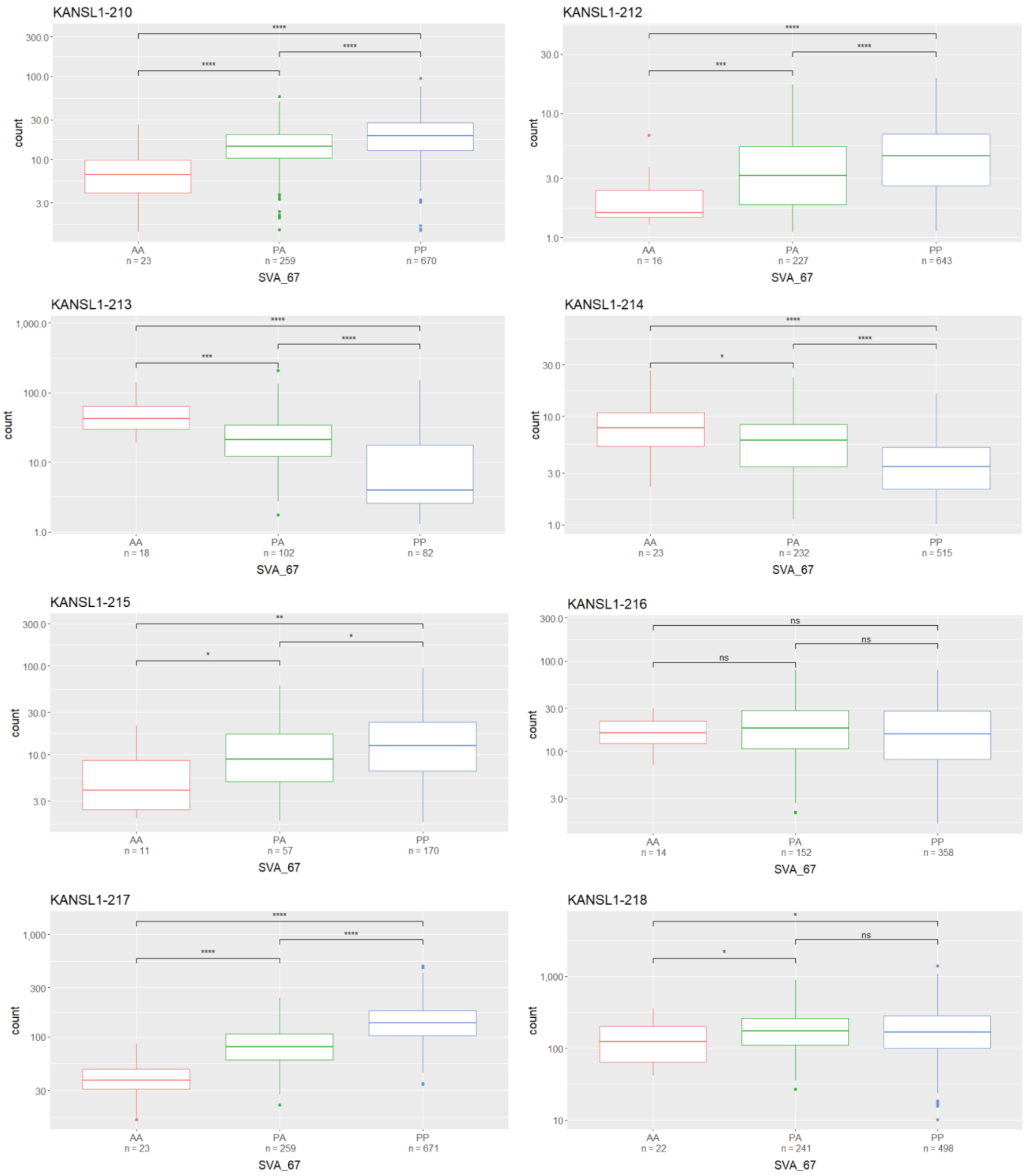

Supplementary Figure 9. Caption page 17.

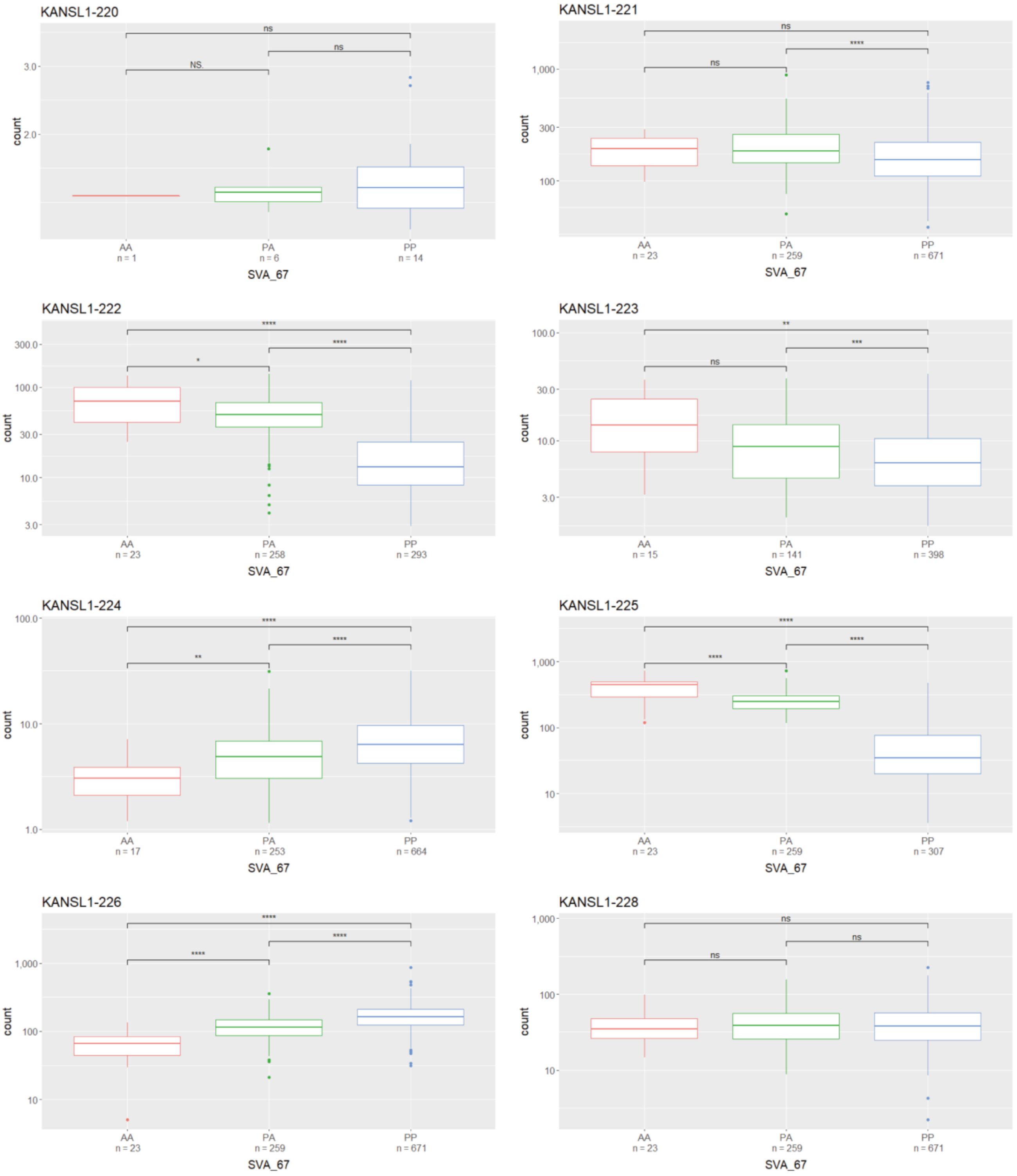

Supplementary Figure 9. Caption next page.

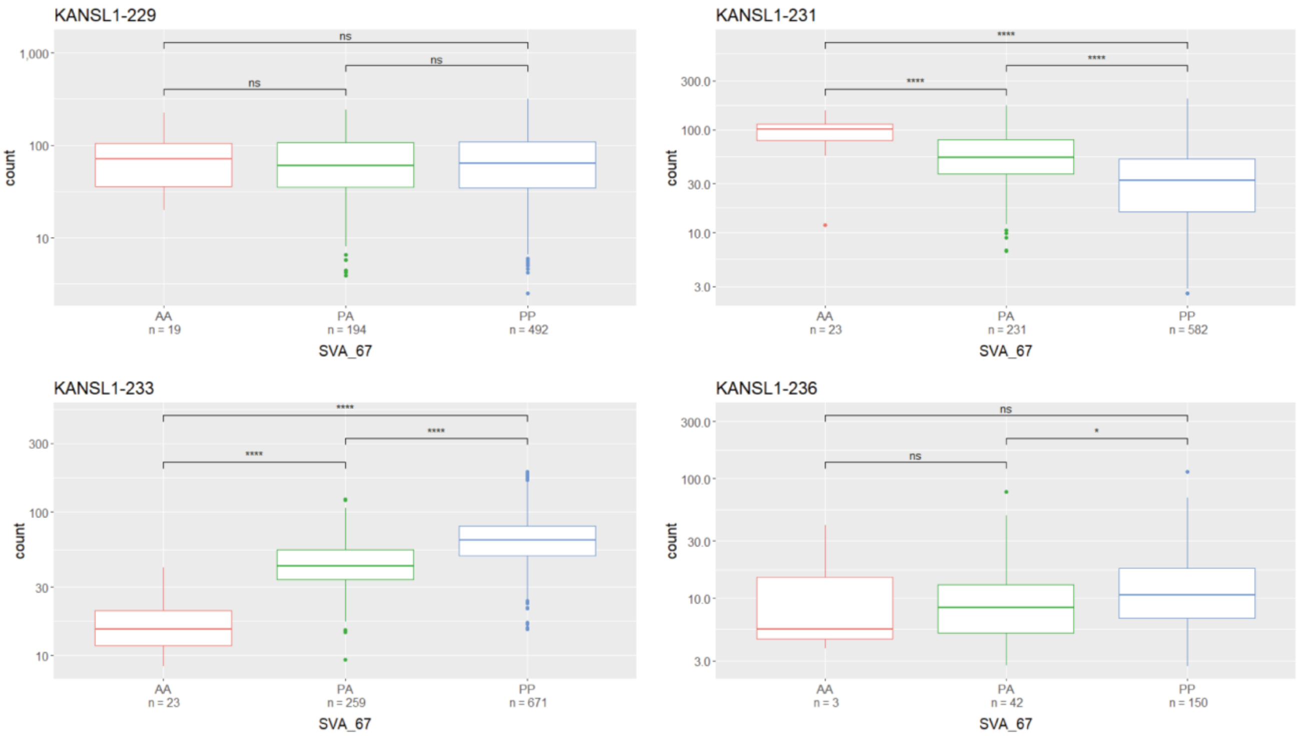

Supplementary Figure 9. Association of SVA_67 genotype with expression of *KANSL1* isoforms using the NYGC ALS cohort. Three different genotypes (*AA*, *PA*, *PP*) were analysed. Wilcoxon test was used to demonstrate statistical significance indicated as asterisks. **P* <= 0.05, ***P* <= 0.01, ****P* <= 0.001, *****P* <= 0.0001, ns > 0.05.

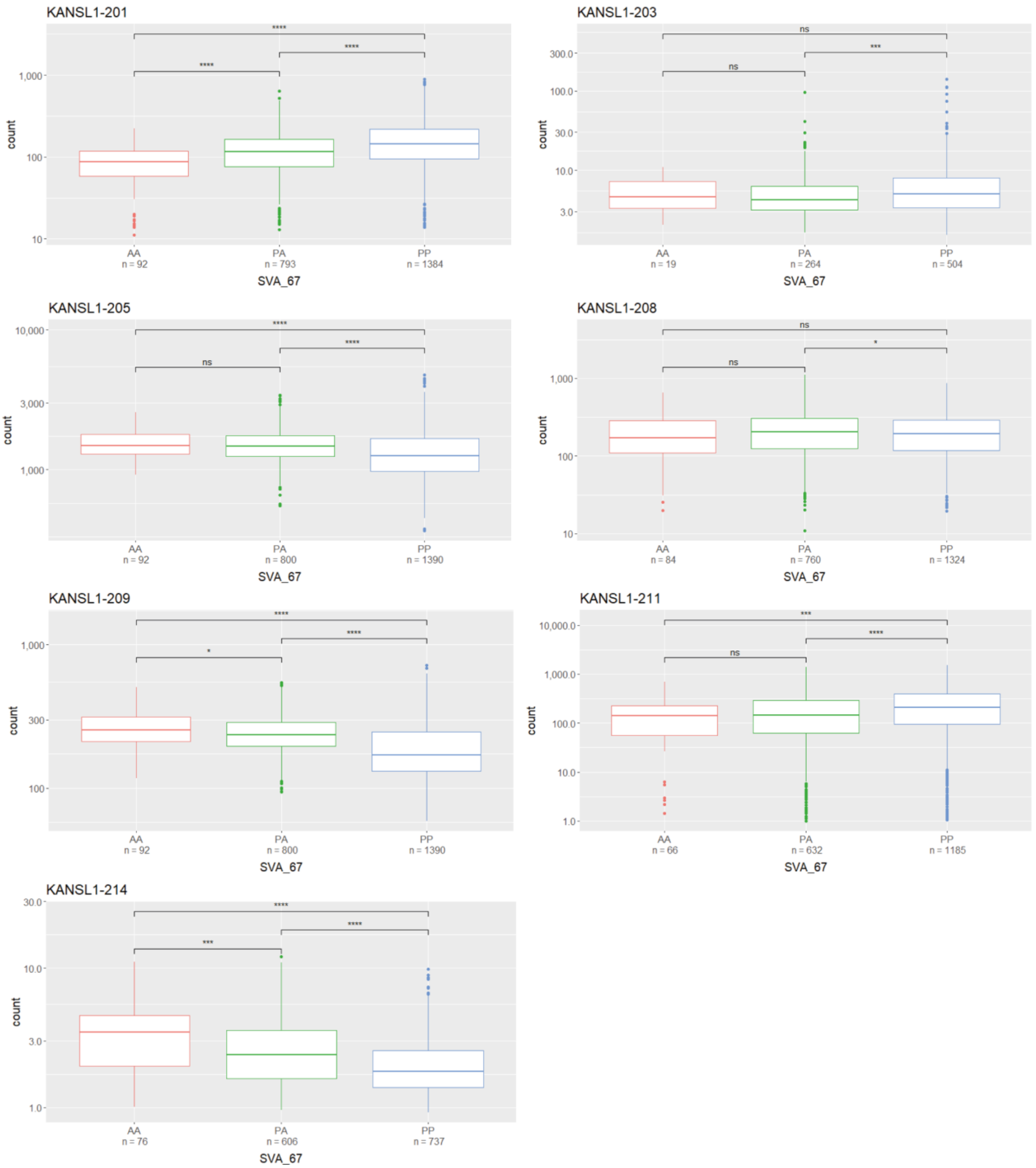

Supplementary Figure 10. Association of SVA_67 genotype with expression of *KANSL1* isoforms using the PPMI cohort. Three different genotypes (*AA*, *PA*, *PP*) were analysed. Wilcoxon test was used to demonstrate statistical significance indicated as asterisks. **P* <= 0.05, ***P* <= 0.01, ****P* <= 0.001, *****P* <= 0.0001, ns > 0.05.
